# Function from Confinement: Ligand-Coated Nanoparticles as Functional Materials

**DOI:** 10.1021/acsnano.5c15028

**Published:** 2025-12-22

**Authors:** Euan R. Kay, Volodymyr Sashuk, Bartosz A. Grzybowski, Fabrizio Mancin, Federico Rastrelli, Verónica Montes-García, Giulio Ragazzon, Paolo Pengo, Lucia Pasquato, Paola Posocco

**Affiliations:** † EaStCHEM School of Chemistry, 7486 University of St Andrews, St Andrews KY16 9ST, United Kingdom; ‡ Institute of Physical Chemistry, 119463 Polish Academy of Sciences, Marcina Kasprzaka 44/52, 01-224 Warsaw, Poland; § Institute of Organic Chemistry, Polish Academy of Sciences, Marcina Kasprzaka 44/52, 01-224 Warsaw, Poland; ∥ Center for Algorithmic and Robotized Synthesis (CARS), Institute for Basic Science (IBS), 50 UNIST-gil, Ulsan 44919, South Korea; ⊥ Department of Chemistry, Ulsan Institute of Science and Technology, 50 UNIST-gil, Ulsan 44919, South Korea; # Department of Chemical Sciences, University of Padova, Via Francesco Marzolo 1, 35131 Padova, Italy; ∇ Institut de Science et d’Ingénierie Supramoléculaires (ISIS), Université de Strasbourg & CNRS, 8 Allée Gaspard Monge, 67000 Strasbourg, France; ○ Department of Chemical and Pharmaceutical Sciences, University of Trieste, Via Licio Giorgieri,1 34127 Trieste, Italy; ◆ Department of Engineering and Architecture, 9315 University of Trieste, Via Alfonso Valerio 6/A, 34127 Trieste, Italy; ¶ Center for Advanced Technologies, Adam Mickiewicz University, Uniwersytetu Poznaskiego 10, Poznań 61-614, Poland

**Keywords:** self-assembled monolayers, functional nanomaterials, nanoconfinement, nanozymes, nanoparticle assembly, nanocatalysis, molecular recognition, sensing, stimuli-responsive, adaptative materials

## Abstract

For nanoparticles stabilized by self-assembled monolayers, the surface-bound molecular species not only modify the core material properties but also provide a handle for interaction with other components, whether they are molecular, nanoscale, or even macroscopic. Importantly, when confined to nanosurfaces, these organic entities exhibit emergent properties that impart unique functionalities to the underlying nanomaterial. In this Review, we examine how these capabilities originate from the structural organization and collective interactions within on-nanoparticle self-assembled monolayers, drawing on examples of quasi-spherical nanoparticles smaller than ca. 8 nm in size. Our focus spans four key categories of function: (i) catalysis and chemical transformation under nanoconfinement, (ii) molecular recognition and sensing, (iii) switching and adaptation, and (iv) programmable nanoparticle assembly. By adopting a systems-chemistry perspective to identify how function is defined by chemical constitution, we elucidate design principles and strategies that we envisage can be broadly applied to a variety of hybrid organic–inorganic nanosystems. We also highlight the current challenges and future opportunities in the field of functional nanoparticles stabilized by self-assembled monolayers. Our aim is to motivate the community to shift toward a perspective in which the organic layer is understood as an active driver of the system functionality rather than a passive component. By harnessing its dynamic and adaptative nature, researchers can design functionally sophisticated and chemically programmable nanomaterials, unlocking unexplored possibilities in active materials, nanocatalysis, molecular recognition, sensing, and delivery.

## Graphical abstract

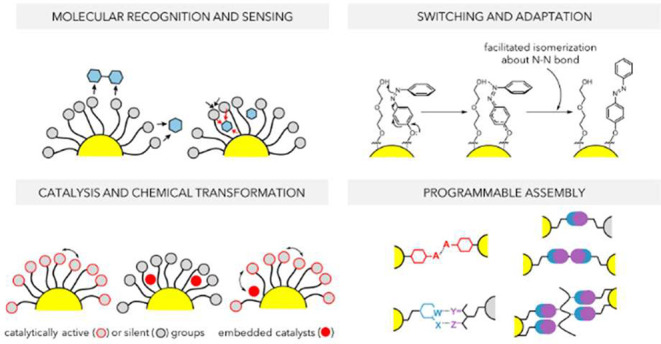

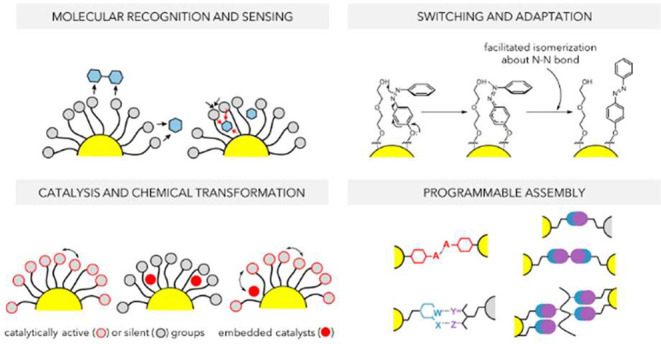

## 1 Introduction

Self-assembled monolayers (SAMs) of small molecules formed on the surface of metal nanoparticles (NPs) serve multiple purposes. In general, SAMs constitute a protective layer stabilizing the NPs against aggregation and conferring colloidal stability in different environments.^1^ Additionally, they enable precise surface modification, allowing for the selective attachment of biomolecules, drugs, or responsive units.^2–7^ SAMs can be tailored for specific biomolecular interactions,^8–13^ facilitating targeted drug delivery and molecular recognition.^13–17^ Beyond biological applications, the nature of the SAM influences the optical (plasmonic) and electronic properties of metal NPs, making them valuable in sensors, photovoltaics, and catalysis.^18–20^ Moreover, SAMs can be engineered to respond to stimuli such as pH, temperature, light, or redox conditions, enabling spatiotemporally controlled functions, such as drug release, catalysis, and signal transduction in biosensors.^21^

The maturity now reached in synthesis, analytical and computational characterization of SAM-protected NPs (SAM@NPs) has set the stage for creating increasingly sophisticated nanosystems, in which multiple and advanced functions can be integrated and executed in response to a specific sequence of instructions. Capitalizing on the knowledge gained over the past years, it is also important to embrace key emerging concepts.

Among these, it has become apparent that nanometer length-scale surface confinement corresponds to a unique environment, fundamentally different from the surrounding bulk solution, that can be exploited to generate distinctive functionality and behaviors.^22^ These confinement effects can involve the entire organic layer or be limited to specific regions, such as loci within the SAM interior or on its periphery, allowing for the coexistence within the nanostructure of multiple distinct areas having unique functional fingerprints. An elegant example of how the surface-confined environment can strongly influence the functional behavior of even a structurally simple SAM@NP was demonstrated by Pillai and co-workers,^23^ who devised a control system based on gold NPs (AuNPs) covered with alkyl thiolates bearing charged end groups. The role of these charged moieties was to “gate” the transport of a charged substrate, 4-nitrophenolate anion, toward the Au surface where it could be reduced to 4-aminophenolate. When the ligand end groups were positively charged (−CH_2_–N­(CH_3_)^3+^, leading to a zeta potential of +24 mV), the negatively charged substrate could pass freely, and the surface catalysis was “ON”, with an apparent rate constant of 0.71 min^–1^; however, when the end groups were negatively charged (−COO^–^ and a zeta potential of −22 mV), the approach of the substrate was hindered by electrostatic repulsion, and the catalysis was “OFF”, with a rate approximately 1 order of magnitude slower, and in fact no catalysis observed over 8 h at the same catalyst loading. Thus, a SAM that electrostatically attracts ions or charged molecules locally enhances the concentration of oppositely charged species at NP surface and within the monolayer compared to the bulk solution. The same principle could be applied to restrict access of like-charged species to the monolayer.

Several factors concur to create unique surface-bound settings, where the chemical and physical properties of ligands in the monolayer may differ from their behavior in bulk solution,^24^ including for instance restricted mobility of the ligand chains due to binding with the metal surface, conformational flexibility limited by crowding by neighboring ligands, and enhanced intermolecular interactions within small solvated volumes. For example, the acid–base behavior of ligands bearing ionizable groups changes significantly compared to when they are free in solution. Similar to the well-known effects in surfactant aggregates,^25,26^ attachment to a nanoscale surface forces the ligands to “communicate” with each other, and a delicate balance of chemical free energy, electrostatic forces, van der Waals interactions, steric effects, and packing constraints governs the local environment around such ionizable groups, ultimately affecting their protonation state. The curvature of the surface also helps define how ionizable groups are positioned in space, further influencing their chemical behavior.^27,28^ When ligands terminate with −COOH groups and bind to AuNPs, their apparent p*K*_a_ shifts to a value between that observed for free ligands in solution (p*K*_a_ ≈ 4.8) and that for monolayer-bound ligands on planar gold surfaces (apparent p*K*_a_ ≈ 10).^28^ As another example, Kay and Posocco^29^ studied SAMs with peripheral reactive groups (hydrazones) positioned at varying distances from the Au core. Using ^19^F Nuclear Magnetic Resonance (NMR) spectroscopy and molecular modeling, they quantified hydrazone reactivity, and showed that reduced conformational mobility of the on-particle ligands decreases the number of productive encounters between surface-confined hydrazones and incoming water nucleophiles, resulting in lower reaction rates compared to the same substrates in solution. Shorter ligands showed a more significant drop in reactivity compared to bulk solution behavior, as their reactive hydrazone ends became buried within a disordered SAM, whereas the more ordered monolayer formed by bundling of longer ligands counterintuitively exhibited higher reactivity because the reactive sites were more exposed to the solvent and the nucleophilic water molecules.

Spatial confinement can also trigger collective behaviors. Indeed, confined environments limit molecular mobility and conformational flexibility, compelling ligands to adopt well-defined orientations and arrangements that promote cooperative interactions and create local supramolecular environments. Intermolecular forces, such as hydrogen bonding, van der Waals forces, and electrostatic interactions, are strengthened due to close proximity. As a result, ligands no longer act independently but instead function in a concerted manner. This interplay between confinement and cooperative interactions gives rise to emergent properties, where the system (the monolayer) as a whole exhibits behavior that is not simply the sum of its individual components (the ligands). As a consequence, functional properties such as molecular recognition, nanocatalysis, signaling, and responsiveness may emerge entirely from the organic monolayer rather than the individual inorganic support or ligand molecules alone.

In recent years, several studies have provided concrete examples of how local environments in SAM@NPs impart functionality. Nonetheless, due to the collective nature of the phenomena involved, function remains challenging to predict based only on monolayer composition, ligand chemistry, and intuition. Additionally, the lack of systematic rationalization highlights the need for a common ground to establish foundational principles that could guide the design of more advanced, ideally multicomponent and multifunctional, SAM@NPs useful for practical applications.

This Review takes the first step in addressing this challenge by adopting a systems-chemistry perspective and analyzing relevant examples from the literature. We aim to establish generalizable principles that guide the design of SAM@NPs with tailored functionalities, focusing on four key areas and the underlying mechanisms driving their functional abilities. (i) *Catalysis and chemical transformation under nanoconfinement*. SAM@NPs naturally form multivalent surface interactions, which can be used to create catalytic hotspots, enabling the design of synthetic enzyme mimics with enhanced efficiency, or able to promote specific reactions. Additionally, the monolayer may serve as a spatial or temporal “gate”, controlling access to catalytic centers, whether at the core or embedded in the monolayer. Regulation mechanisms rely on a careful selection of the monolayer components and the implemented regulation strategy. (ii) *Molecular recognition and sensing*. Platforms for molecular recognition and sensing exploiting SAM@NPs are multivalent and cooperative systems whose affinity and selectivity toward selected molecules or groups of target analytes can be tailored by designing *ad hoc* the monolayer constituents. Examples targeting a variety of (bio)­recognition moieties have already been demonstrated, including oligonucleotides, antibodies, peptides, proteins, microorganisms, drugs, and other small molecules. Strategies for molecular recognition and sensing are classified and described in the context of instructive examples, with particular attention given to systems in which ligand choice and nanosurface confinement are together essential for endowing SAM@NPs with functional capabilities in response to particular analyte(s). (iii) *Switching and adaptation*. Suitable ligands enable SAM@NPs to switch their properties in response to external stimuli such as pH, light, and electrons. Switchable ligands undergo instructed structural or chemical changes, which in turn precipitate changes in surface organization or local properties, ultimately altering the NP behavior. Due to the collective behavior of the SAM@NP system, switchability is closely linked to adaptability,^30,31^ the capacity to adjust to environmental changes (alternating energy landscape upon the application of the trigger/countertrigger), often leading to emergent behaviors. Adaptability is an essential element in bioinspired active matter. (iv) *Programmable NP assembly*. The features of the surface-bound ligands can be designed to program structural, compositional and dynamic control over SAM@NP assembly. Molecular length-scale features are translated through covalent and noncovalent intra-SAM and inter-SAM interactions to determine composition, arrangement and dynamic behavior of assemblies spanning multiple length scales. This chemically informed approach facilitates the construction of NP superstructures under equilibrium or out-of-equilibrium conditions, with tailored properties that can be responsive to external inputs.

This Review focuses on SAM@NPs with core (primarily Au) dimensions generally ≤8 nm. In this size range, surface curvature has a critical influence on defining local monolayer environments. As core size increases, the effect of curvature diminishes, so that beyond ∼8 nm, surface effects approach those of a flat substrate.^32^ The majority of examples constitute quasi-spherical Au cores stabilized by monolayers derived from alkylthiol molecules. Indeed, AuNPs can now be prepared in a quite reproducible and controllable manner.^33,34^ The Au cores are relatively chemically inert, while exhibiting a strong affinity for thiols.^35^ This allows for the formation of stable and consistent SAMs, decoupled from the core preparation, in a range of solvents. Monolayers on AuNP cores can be further modified through various synthetic strategies.^4,6,16,36,37^ Compared to other metal cores, these properties make SAMs on Au not only widely studied but also an ideal model system. The confinement effects we discussed here are primarily a function of the nanosurface-confined SAM itself, meaning that the same principles can be extended to any other nanoscale scaffold on which structurally well-defined monolayers can be prepared.

During the preparation of AuNPs, chiral ligands can be grafted onto achiral AuNP cores to form chiral monolayers as discussed later (see Section 2); alternatively, chiral ligands or chiral inducers may be employed to generate chiral Au cores, where chirality arises from the asymmetry of the nanocrystal facets. This second class of materials, characterized by high-index facets and chiroptical activity at the plasmon resonance wavelength, is gaining increasing attention in plasmonics, sensing, and biomedical applications.^38–41^ Yet, chiral nanomaterials such as chiral AuNPs lie beyond the scope of this review and are discussed in recent themed collections.^42,43^

Confinement effects also arise in other NP surface-stabilizing architectures, including polymer encapsulated,^44^ surfactant stabilized^45–47^ and biomolecule bound systems.^48,49^ However, SAM@NPs constructed from small abiotic molecular components, anchored on the NP core through kinetically stable interactions, hold unique potential for precisely engineering NP surface chemistry. Informed by increasingly detailed insights on multilength scale structure and dynamics available from advanced analytical and computational methods, it has become possible to leverage the distinctive nanosurface-confined environment to achieve remarkable functional behaviors.

## 2 Catalysis and Chemical Transformation under Nanoconfinement

### 2.1 Mimicking Enzymes

One of the fundamental yet, arguably, underappreciated differences between macroscopic pieces of metal and metal NPs is their surface curvature—it is minimal for the former but for the latter assumes values high enough to modulate molecular organization and function. In particular, curvature controls the degree to which SAMs of ligands adsorbed onto NPs “fan out” from the surface. On larger NPs, the curvature is low and the monolayers are tightly packed, strongly passivating the metal surface, limiting molecular conformational freedom and restricting the ability of external species to enter the monolayer environment. As NPs become smaller, the increased curvature allows ligands greater conformational flexibility, creating free volume in-between and allowing other molecules to penetrate the monolayer. These incoming molecules can experience catalytic effects within the SAM environment. With SAMs comprised of more than one type of ligand, the possibilities for molecular-level engineering of the system behavior are further extended. For instance, only some ligands can be functional (say, for instance, stimuli-responsive or/and catalytic), while others (spectator or background ligands) can serve to space out these functional units to a desired degree and/or to surround them within premeditated molecular environments. In addition, the spontaneous ligand clustering on the NP surface may offer opportunities to further increase the interligand space, providing a finite number of internal voids. Capitalizing on these characteristics, one can think of creating enzyme-like cavities in which active centers are within ”pockets” whose shapes and dimensions are dictated by NP size (via particle curvature) and ligand–ligand interactions (through clustering or specific interligand interactions), whereas utilitarian properties of the pocket are encoded in the surrounding functional groups (Figure 1).

**Figure 1:**
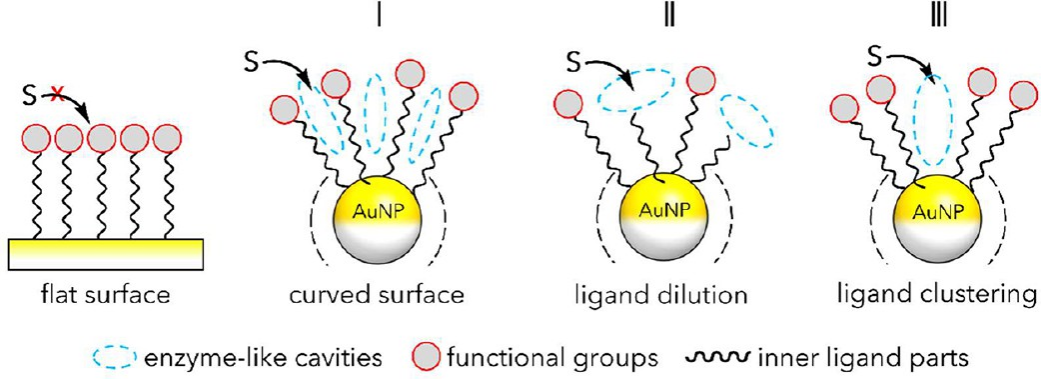
Difference between SAMs on flat and curved surfaces. On a curved surface, the formation of interligand voids within the organic monolayer—accessible to external molecules or substrates (S)—is possible (I) thanks to the fanning of ligands moving away from the curved surface. The size and shape of these pockets can further be modulated by dilution of functional ligands with background ones (II) and/or through ligand clustering (III) leading to self-organized nanoreceptors.

Early examples of catalysis by SAM@NPs aimed at developing catalysts that could be easily recovered from the reaction medium while—owing to the flexibility of the ligands in the monolayer—maintaining similar performance to the free catalyst in solution in terms of chemical yield and stereoselectivity. To this aim, octanethiolate-protected AuNPs were functionalized via a place-exchange process with thiolates bearing end-group catalysts or metal chelators already known for their catalytic activity. Studies of mixed monolayers formed in this way demonstrated that catalytic centers on the outer surface of the SAM@NPs retain their catalytic activity^50,51^ or even exhibit higher activities compared to analogous molecular (non-NP-bound) catalysts.^52,53^

To behave as a synthetic enzyme mimic, a NP has to interact with the substrate, stabilizing the transition state, thus facilitating alternative activation pathways. The catalytic site may exhibit distinct solvation properties and local pH compared to bulk solution and the functional groups should be properly oriented and maintained in close proximity long enough to enable kinetic cooperativity. The resemblance to enzymatic behavior led Pasquato and Scrimin to classify systems exhibiting these features as *nanozymes* (Figure 2, *approach I*).^54,55^

**Figure 2:**
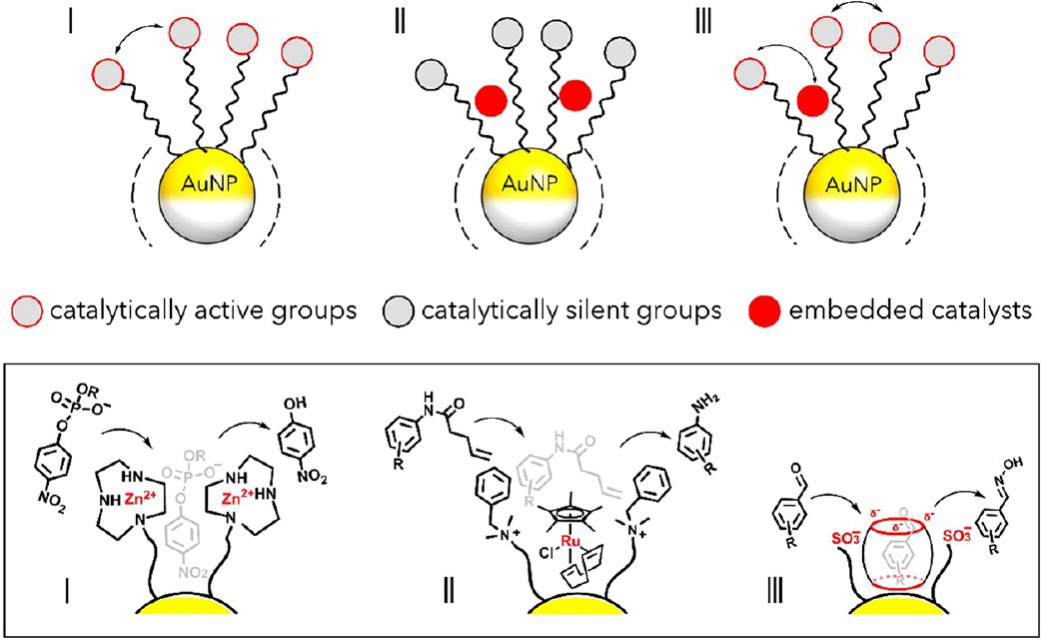
General approaches to nanozyme design: (I) through interactions between proximal functional groups on surface-neighboring attached ligands; (II) via embedment of a molecular catalyst within a catalytically *inactive* monolayer; (III) through embedment and interaction of a molecular catalyst with a catalytically *active* monolayer. Box illustrating examples of I (from ref 56), II (from ref 57), and III (from ref 58).

In a pioneering study, *N*-methylimidazole-functionalized AuNPs were shown to catalyze the cleavage of a carboxylic acid ester.^59^ By assessing catalyst activity across different pH values of bulk solution, the highest activity was observed at a pH corresponding to the p*K*_a_ of the *N*-methylimidazole units. This finding demonstrated that effective catalysis requires the simultaneous operation of general acid and general base or nucleophilic mechanisms, implying intramonolayer cooperativity between two *N*-methylimidazole units. Similarly, Salvio reported an artificial phosphodiesterase based on SAM@NPs.^51^ Their system featured guanidinium-functionalized ligands and was tested for the transesterification of 2-hydroxypropyl *p*-nitrophenyl phosphate (HPNP). The reaction rate versus pH profile displayed a bell-shaped curve, reinforcing the operation of a cooperative catalytic mechanism involving the concerted action of multiple ligands—a neutral guanidine serving as a general base and a protonated guanidine acting as a general acid.

More complex nanozymes, comprised of peptide-decorated AuNPs, further demonstrated the role of cooperative interactions between functional groups characterized by different p*K*_a_ values in facilitating esterase-like activity in model systems. For instance, SAM@NPs featuring a carboxylate from phenylalanine and an imidazolium ion from histidine achieved a 300-fold acceleration in model ester hydrolysis at pH < 7, a condition where the monomeric peptide alone was inactive.^60^ The Scrimin group reported an example of dodecapeptide-functionalized AuNPs^61^ that not only display kinetic cooperativity^62^ between different functional groups within the peptide monomer, but were also able to bind a hydrophobic substrate, with formation of a hydrophobic catalyst-acyl intermediate, that reduces the hydration state of the catalytic site. This decrease in hydration enhances the nucleophilicity of the imidazole units, leading to an apparent p*K*_a_ value 1.1 units higher in the AuNP microenvironment with respect to an analogous model peptide in solution. Consequently, the hydrolysis rate of the catalyst-acyl intermediate is slowed down, causing the accumulation of the intermediate. As a result, catalytic activity is selectively modulated for hydrophobic substrates. The above examples, along with others,^63^ highlight how ligand confinement can generate catalytic microenvironments reminiscent of enzyme active sites, promoting kinetic cooperativity, substrate binding via hydrophobic interactions, and substrate selectivity (Figure 3A,B).

**Figure 3:**
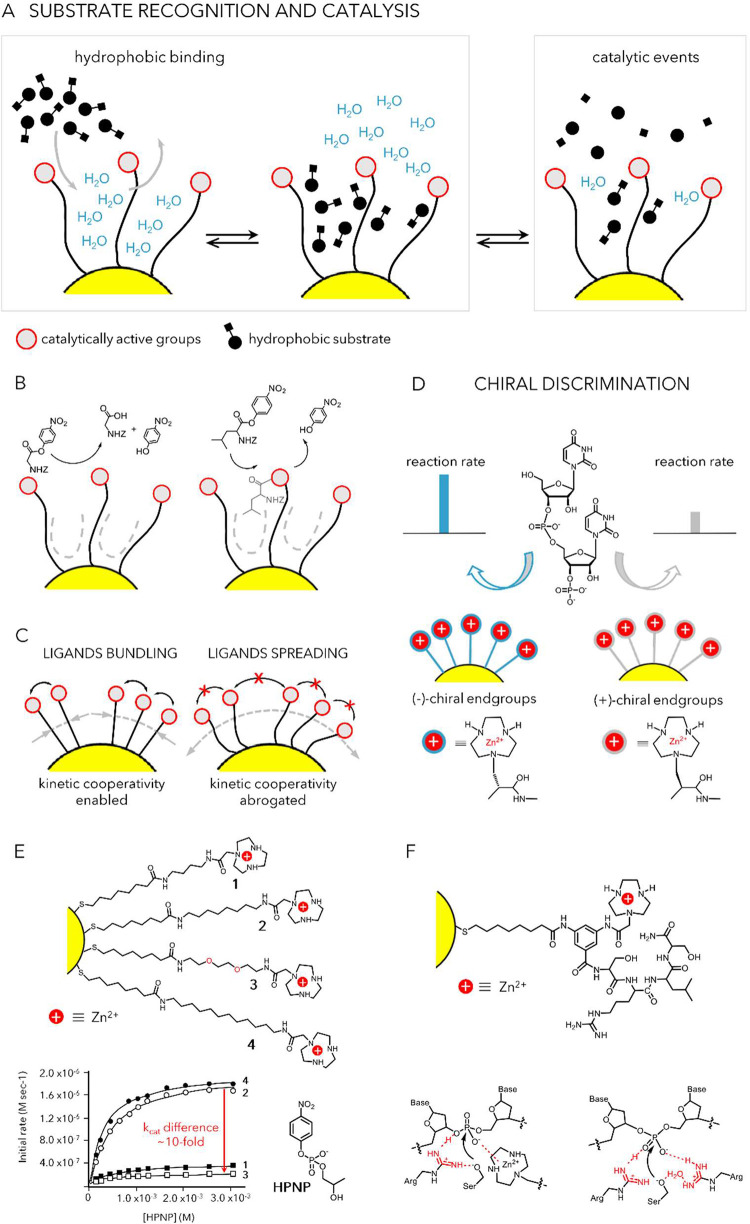
Mimicking enzyme features and activity by SAM@NP. (A) Molecular recognition by hydrophobic interactions. (B) Substrate selectivity due to *in operando* responsiveness of the monolayer hydrophobic environment.^61^ (C) Kinetic cooperativity driven by bundling of ligands. (D) Chiral discrimination by enantiomeric SAM@NP. Adapted with permission from ref 64. Copyright 2016 Wiley-VCH GmbH. (E) Enhanced *k*_cat_ due to the increased hydrophobicity of SAM@NP. Reprinted with permission from ref 56. Copyright 2014 American Chemical Society. (F) Participation of multiple functional groups in DNA hydrolysis by SAM@NP.^65^

Cooperativity between functional groups in the side chains of amino acids or their analogues is one of the strategies for mimicking enzyme function. In many cases, Nature has evolved enzymes in which key reactive centers contain one or more metal ions that act cooperatively. SAM@NPs have proven to be excellent scaffolds for replicating this feature, while also serving as NP-based catalysts that enable the preorganization of bimetallic centers^53^ (Figure 3C). The occurrence of a catalytic action involving proximal metal centers in the intramolecular transesterification of the RNA model HPNP by nanozymes comprising SAM@NPs bearing a triazacyclononane (TACN)-functionalized amino acid was clearly demonstrated by the sigmoidal dependence of the observed rate constants as a function of the loading of Zn^2+^ ions.^54^ Furthermore, introducing the TACN moiety at the side chain of an enantiopure amino acid enabled Prins and co-workers to report the first example of chiral discrimination in the nanozyme-catalyzed transesterification of a HPNP analogue and of the dinucleotide UpU (Figure 3D).^64^

These reactions require intramolecular nucleophilic attack of a hydroxyl group on the phosphorus atom of a phosphate diester. It could therefore be anticipated that increasing the hydrophobicity at the reaction site would enhance catalytic performance. Indeed, this was found to be the key factor behind the remarkable differences in reactivity toward HPNP cleavage among a series of TACN-functionalized nanozymes, in which the TACN unit was systematically positioned at varying distances from the NP surface (Figure 3C,E).^56,66^ Elongating the carbon chain of the ligands, while potentially introducing greater conformational flexibility, did not hinder substrate binding. Instead, the longer alkyl chains created a desolvated, low-polarity environment, which enhanced the reactivity of the NP-bound substrate. Additionally, it was later shown by molecular dynamics (MD) simulation that hydrophobic clustering of long alkyl ligands increases the likelihood of forming bimetallic sites.^66^ It thus appears that moving the catalytic center within the monolayer along the radial direction may be useful in tailoring catalytic activity. In that regard, Koksch and co-workers^67^ reported that the positioning of a histidine catalytic unit within the peptide-monolayer relative to the Au core surface significantly impacts both the hydrolytic activity of *p*-nitrophenyl esters of protected amino acids and the substrate selectivity of peptide functionalized AuNPs. Specifically, NPs with the histidine residue closer to the Au surface exhibit greater efficiency in cleaving highly hydrophobic ester substrates, while less hydrophobic substrates undergo faster hydrolysis when the histidine residue is located in the intermediate region of the monolayer.

As discussed above (Figure 3D and related example),^64^ chirality can be leveraged to endow the monolayer with enantioselectivity and the ability to form chiral, catalytically active pockets. In that example, the functional groups responsible for the formation of the chiral monolayer were also involved in the catalytic transformation. Chiral ligands can alternatively serve to create a chiral environment in which the metal surface of the NP acts as the catalytic center. Ding, Wang and co-workers^68^ showed that AuNPs coated with single-stranded DNA preferentially bind and oxidize l-glucose, whereas double-stranded ligands favor d-glucose, reflecting different origins of enantioselectivity arising from nucleotide stacking and helical organization. The same authors further demonstrated that the catalytic selectivity can be responsive to an external stimulus (see also Section 4). The enantioselectivity was reversed at low pH on account of folding of the DNA single-stranded into an i-motif structure. To fine-tune this pH-responsive behavior, an auxiliary strand was introduced to form a duplex at neutral pH and dissociate upon i-motif formation, allowing switching between stereoselective and racemic catalysis according to pH. Chirality transfer is not limited to chemical reactions within the monolayer; it can propagate into the surrounding medium, producing both short^69^—and long^70^—range effects.

Kinetic cooperativity between metal centers and amino acid side chains within the protecting monolayer may turn SAM@NPs into efficient monometallic catalysts for DNA hydrolysis. Indeed, AuNPs featuring a passivating monolayer composed of a TACN-Zn^2+^ complex, a guanidinium ion (from an arginine residue) and a primary alcohol (from a serine residue) efficiently cleave the supercoiled plasmid pBR322 with a half-life of 50 min, under physiological conditions, remarkable result, considering that the natural half-life of DNA is approximately 31 million years. Mechanistic analyses aided by MD simulations are consistent with the hypothesis that the metal center coordinates the phosphate group, while the guanidinium could help in stabilizing the developing charge on the phosphate, while also decreasing the p*K*_a_ of a serine residue present in the peptide proximal to the metal center making it a better nucleophile (Figure 3F).^65^

As seen in the examples above, proximity of catalytic moieties can be crucial for optimal nanozyme performance. When functionalized thiolates are combined with inactive ones, self-organization may arise leading to distinct ligand spatial arrangements, proximity behaviors, and monolayer morphologies. Among others,^71^ Scrimin and Prins studied such effects on a SAM@NP nanozyme experimentally and theoretically, examining the catalytic efficiency for HPNP transphosphorylation in the presence of Zn^2+^.^72^ The reaction was known to occur only in the presence of a bimetallic catalytic site. Using a TACN-functionalized thiol blended with an oligoether-terminated thiol, they found that the catalytic rate constant (*k*_cat_), normalized for the number of metal centers, increased with the catalytic thiol fraction up to 0.4 and then plateaued. Their theoretical model suggested a random ligand distribution, contrasting with a phase-segregated clustering that would have resulted in a *k*_cat_ constant with monolayer composition.

A distinct approach to nanozymes has been pioneered in recent years by the Rotello group. Rather than relying on ligands bearing catalytic moieties grafted onto the NP surface or assembling catalytic units directly on it, their strategy involves the embedding of catalytically active transition metal complexes (TMCs) within the NP monolayer (Figure 2, *approach II*).^73–78^ This method offers several advantages. First, TMCs can be selected to catalyze reactions that typically do not occur in biological environments, enabling the development of bioorthogonal nanozymes. Additionally, embedding TMCs within the monolayer preserves their catalytic activity even in aqueous media, where they would otherwise decompose. Moreover, since the encapsulation of hydrophobic, uncharged TMCs does not alter the NP surface groups, these can still participate in molecular recognition via electrostatic interactions or supramolecular host–guest binding, potentially allowing for environmental responsiveness.

The use of bioorthogonal nanozymes has been explored for prodrug and profluorophore activation without interference from endogenous enzymes, offering an alternative strategy for therapeutic and imaging applications.^73^ Specifically, leveraging NP surface charge modulation to control cellular uptake, cell-penetrating cationic NPs loaded with Cp*Ru­(cod)Cl (Cp*=pentamethylcyclopentadienyl; cod = cycloocta-1,5-diene) TMC, a catalyst for deprotecting the allyloxycarbonyl (Alloc) amino-protecting group, were used for intracellular catalysis. In contrast, “stealth” zwitterionic catalytic NPs, which are not internalized by cells, restricted catalysis to the extracellular space. The spatial localization of the two nanozymes was first confirmed using cell-permeable and cell-impermeable profluorophores, which emitted distinct signals upon catalytic cleavage of the allylcarbamate protecting group. Furthermore, the viability of HeLa cells treated with an allylcarbamate-protected doxorubicin prodrug was significantly reduced when exposed to cationic, cell-permeable TMC-loaded nanozymes, whereas the noncell-penetrating zwitterionic nanozymes were much less effective. Subsequent studies highlighted the protective role of the hydrophobic inner shell of water-dispersible AuNPs in stabilizing the water-sensitive Cp*Ru­(cod)­Cl TMC within the monolayer.^74^ While the free catalyst loses activity within 4 h in aqueous media, its incorporation into the NP monolayer preserves full catalytic function over the same period.

Bioorthogonal nanozymes enable localized activation of prodrugs (and imaging agents), minimizing systemic toxicity and ensuring targeted drug release during administration. The prodrug is administered systematically but transformed to its active form only locally by injection of the bioorthogonal nanozymes in the tumor mass.^75^ One strategy involves injecting positively charged nanozymes directly into the tumor, where they persist active for days due to strong interactions with cell surfaces, allowing sustained prodrug activation.

An alternative approach for targeting was demonstrated by using macrophages to transport cationic AuNPs incorporating a Pd­(II) catalyst, which deprotects propargyloxycarbonyl groups.^76^ Macrophages internalize these nanozymes while retaining viability and migratory ability over days. When cocultured with HeLa cells in the presence of a propargyl-protected 5-fluorouracil prodrug, the macrophage-internalized nanozymes activate the drug, which then diffuse to the cancer cells. Another approach utilized red blood cells (RBCs) to deliver TMC-nanozymes to bacterial biofilms. Grafting positively charged nanozymes onto RBCs facilitate their transport. Upon RBC hemolysis by bacterial toxins, the nanozymes are released and catalytically activate moxifloxacin, from its protected form, demonstrating a potential strategy for antimicrobial therapy.^77^

The availability of surface groups not directly engaged in catalytic activity provides an opportunity to fine-tune SAM@NP interactions with target environments. One approach to targeted delivery for imaging purposes involves nanozymes with pH-responsive end groups that switch between zwitterionic and positively charged states.^78^ In their zwitterionic form, these nanozymes do not significantly penetrate biofilms. However, the acidic biofilm environment converts the ligands to their cationic state, leading to accumulation within the biofilm. Once retained, these nanozymes can activate pro-fluorophores, generating fluorescence signals that provide optical imaging of infected sites.

The third approach to nanozyme preparation combines elements of the first two strategies, leveraging the catalytic properties of both covalently grafted functional groups and noncovalently bound molecular species (Figure 2, *approach III*). The Sashuk group^300,58^ has demonstrated that the catalytic performance of cucurbituril macrocycles, recognized organo-catalysts, can be considerably enhanced upon their interactions with AuNPs coated with sulfonic acid groups in a model acid-catalyzed oxime formation reaction. The rate enhancement was rationalized as arising from the embedment of the macrocycle molecules within monolayer voids opened by ligand clustering and the synergistic action of these hydrophobic cavities with the ligand-attached sulfonic acids, which lower the local pH, yield a supramolecular assembly the authors termed a “suprazyme”, where both the macrocycle and NPs contribute to enzyme-like properties of the system.

### 2.2 Regulating Catalysis within the Monolayer

Apart from facilitating chemical reactions or enabling specific ones, one can use the organic ligands on SAM@NPs for spatio/temporal gating of molecules diffusing into the monolayer, leading to selective incorporation from bulk solution and regulation of SAM@NP catalytic activity. The ability to accept or deny incoming substrates within the monolayer is crucial in this context. This can be achieved either by exploiting hydrophobic and/or intermolecular interactions of the small molecules with the monolayer, which then acts as a pseudophase preconcentrating the reactants^79–82^ or by leveraging more sophisticated strategies, allowing for immediate or temporal control of substrate access.

Immediate control involves the introduction of functional groups into the monolayer that permanently act as molecular “gatekeepers” at the interface. These groups regulate access to the NP-bound catalytic sites by exploiting electrostatic interactions or/and steric (mis)­matches between the substrate and the monolayer environment (Figure 4, *approach I* and *II*). For instance, AuNP surfaces coated with positively charged monolayers are far more efficient in catalytic reduction reactions involving borohydride anions compared to those with negatively charged coatings (Figure 4Ia).^23^ In a related study, Grzybowski and colleagues showed that charged functional groups on the NP surface could also control the entry direction of mutually reactive ionic species into the monolayer.^83^ In their system, positively charged groups surrounding copper catalytic sites facilitated the passage of negatively charged azide and alkyne substrates, dictating substrate selectivity in a model Huisgen cycloaddition (Figure 4Ib).

**Figure 4:**
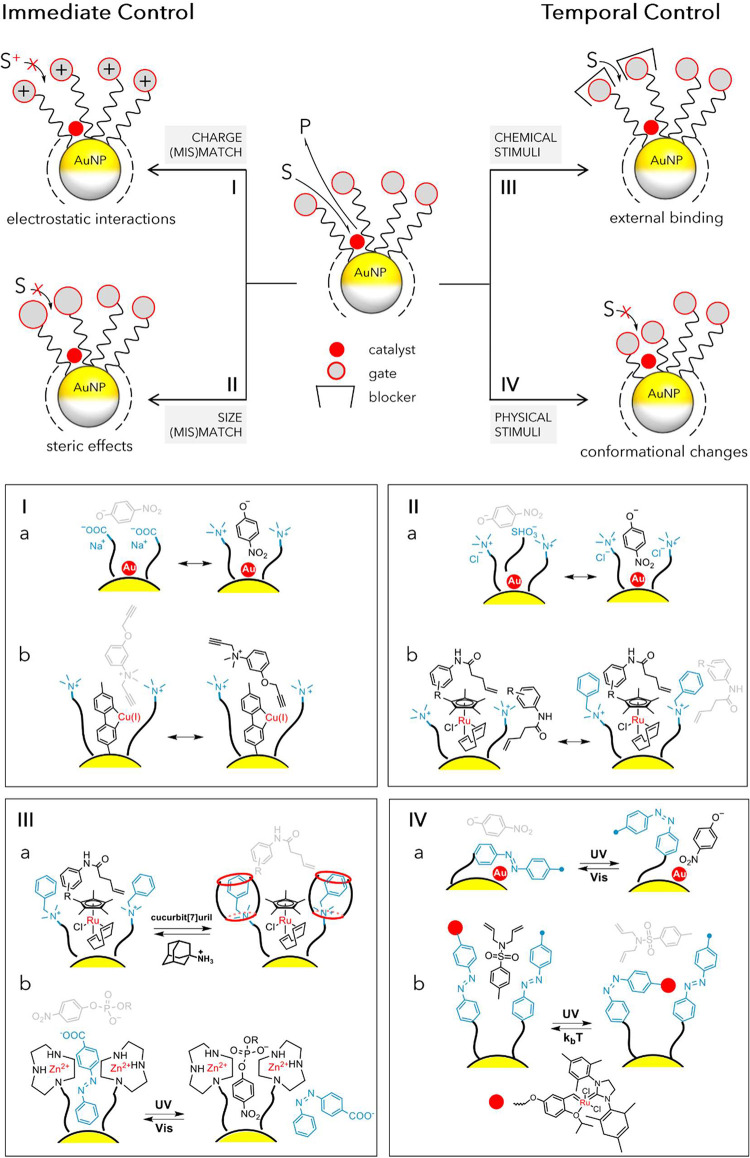
General strategies for regulating catalysis within SAM@NPs (top panel) and representative examples (bottom panels). These approaches can be employed either independently or in combination, utilizing different stimuli and types of interactions. The active site may consist of a covalently or noncovalently bound molecular catalyst, or the metal surface itself. The gate group may be part of the ligand or a monolayer-associated counterion. (I) Charge (mis)­match: Ia from ref 23; Ib from from ref 83. (II) Size (mis)­match: IIa from ref 84; IIb from ref 85. (III) Chemical stimuli: IIIa from ref 57; IIIb from ref 86. (IV) Physical stimuli: IVa from ref 87; IVb from ref 88.

Remarkably, when the dialkyne substrate bore the same charge as the monolayer, the reaction still proceeded, but only the triple bond positioned farther from the charged group was able to penetrate into the monolayer, resulting in site-selective transformation. Furthermore, Maiti and co-workers took advantage of various binding strengths of mono- and multicharged ions with an oppositely charged NP surface, accompanied by changes in local pH, to attain catalytic preference for distinct chemical reactions.^89^ Subsequent work by Grzybowski and his team later revealed that the size of counterions around the charged monolayer is equally important for catalysis efficiency as the charge complementarity with the substrate (Figure 4IIa).^84^ They showed that bulky counterions could block access to the active NP surface and effectively suppress catalysis in the reaction between negatively charged nitrophenolate anions and a borohydride reductant. Importantly, the activity of the NPs could be restored in situ by replacing bulky ions with smaller ones, hinting at the possibility of temporal regulation of catalysis through external control. In another example, tuning the bulkiness of functional groups at the monolayer interface could also modulate monolayer compaction and thus the activity of an embedded organometallic catalyst. Small surface groups promoted monolayer dilation, which facilitated extensive substrate adsorption, ultimately leading to substrate-induced inhibition. In contrast, bulky hydrophobic moieties enabled dense packing and yielded classical Michaelis–Menten kinetics in a model deprotection reaction (Figure 4IIb).^85^

Similarly, Murphy and Baker^90^ demonstrated that a dodecanethiol monolayer markedly enhances the selectivity and stability of AuNP electrocatalysts used for electrochemical CO_2_ reduction to CO, compared to both bare AuNPs and polycrystalline bulk Au. Notably, while the monolayer does not substantially impact the efficiency of CO_2_ reduction, it effectively prevents the electrodeposition of adventitious metal ions, which would otherwise lead to catalyst deactivation. This occurs because hydrated metal ions cannot penetrate the hydrophobic monolayer to reach the catalytic Au surface. This insight allowed the authors to develop an electrode configuration that, under working conditions using ambient water as the electrolyte solvent, achieved a CO yield 100 times higher than that of polycrystalline Au.

While the strategies discussed above rely primarily on permanent features of the monolayer, temporal control can be achieved through external inputs that modulate access to catalytic sites in real time. This includes approaches based on chemical blocking agents (Figure 4—*approach III*), as well as more sophisticated systems incorporating conformationally responsive ligands or photoswitches, as illustrated in Figure 4—*approach IV*.

Host–guest recognition has been extensively used to implement chemical gating mechanisms. Rotello and co-workers demonstrated this concept by designing nanozymes with benzylammonium-functionalized surfaces, where a catalytically active TMC was embedded within the monolayer. Capping the benzylammonium groups with cucurbit[7]­uril (CB[7]) blocked catalytic activity by restricting substrate access. Introducing 1-adamantylammonium chloride as a competitive guest displaced CB[7] restoring catalytic function (Figure 4IIIa).^57^ Building on this approach, the same group developed a noncompetitive inhibition strategy using enhanced green fluorescent protein (EGFP) to regulate nanozyme activity. The protein binds to the cationic nanozyme surface, physically hindering substrate access and reducing catalytic efficiency. Since EGFP binding affinity decreases at high ionic strength, adjusting this parameter provides a tunable mechanism for modulating nanozyme activity.^91^ Further work showed that nanozyme activity could also be controlled by thermally modulating interactions within the monolayer interior.^92^

A related strategy was employed by the Koksch group, who used a catalytically silent, complementary peptide to reversibly block access to a catalytically active peptide immobilized on the NP surface, thereby inhibiting an ester hydrolysis reaction.^93^ The catalytic peptide lacked a defined secondary structure when grafted onto the Au core. However, upon addition of the complementary peptide, a heterodimeric α-helical coiled-coil structure was induced, resulting in the inhibition of catalytic activity through structural reorganization.

To overcome the need for continuous addition of chemical blockers—and the associated accumulation of chemical waste—photoswitchable molecules offer a powerful alternative, enabling reversible and remote-controlled regulation of catalysis. The Prins group demonstrated this by employing anionic azobenzene (AB) derivatives that undergo reversible photoisomerization between a compact *cis* form and an extended *trans* form. Present in solution, these molecules competed with negatively charged substrates for access to a catalytic monolayer composed of cationic zinc complexes.^86^ The two isomers exhibited distinct affinities: the bulky *cis* form was unable to efficiently penetrate the monolayer, leaving the catalyst accessible, while the slimmer *trans* form intercalated into the monolayer, effectively blocking substrate access and inhibiting the reaction (Figure 4IIIb). Embedding the system in a gel matrix further enabled spatial control over catalysis, demonstrating the potential for spatiotemporal regulation.^94^

Dai and co-workers designed AB-containing peptide chains bearing imidazole pendants, which reversibly bound to β-cyclodextrin (β-CD)-capped AuNPs in a photoswitchable manner because only the *trans*-isomer can form a stable host–guest complex with the surface-bound β-CDs.^95^ In the *trans* form, the chains associated with the AuNP surface exhibited functional group cooperativity that promoted ester hydrolysis. Upon UV-induced isomerization to the *cis* form, the peptides lost this interaction and desorbed from the surface, resulting in decreased catalytic activity. In a follow-up study, the same group exploited this switchable binding to enable a light-regulated cascade reaction, in which the hydrolysis product, 4-nitrophenol, was further reduced to 4-aminophenol by catalysis on the disassembled AuNP cores.^96^ This work provides a rare example of multistep catalysis driven by light-responsive transitions between distinct assembly states of a single supramolecular catalyst.

In addition to using photoswitches in solution, NP activity can also be regulated by permanently attaching AB units to the surface, leveraging the geometric and dipole moment changes that occur upon isomerization. One of the pioneering examples of using monolayer-based regulation was developed by the Grzybowski group,^97^ who employed AuNPs coated with a mixture of aliphatic amines and thiols terminated with AB units. In this system, the amines served as inert spacers controlling the surface concentration of the azo photoswitches. Under UV irradiation, the AB residues isomerized to their *cis* forms, increasing their dipole moments. This change induced dipole–dipole interactions between NPs,^98^ triggering aggregation in nonpolar media. The resulting aggregates limited substrate access and slowed a hydrosilylation reaction catalyzed at the Au surface. Subsequent irradiation with visible light reversed the isomerization, leading to disaggregation and reactivation of the catalyst. This reversible “ON/OFF” switching cycle could be repeated multiple times.

Knecht and colleagues creatively advanced this concept by utilizing conformational changes triggered by the isomerization of AB. By incorporating azo fragments into peptide ligands, they harnessed light to induce the rearrangement of the obtained bioorganic monolayer, modulating the exposure of the underlying AuNP surface to 4-nitrophenol from bulk solution and thus controlling its reduction rate (Figure 4IVa).^87^ Subsequent studies by the same research group expanded this strategy by varying the metal core composition,^99,100^ ligand shell structure,^101^ and positioning of azo residues within peptide sequences,^102^ while also providing structural insights into the NP-ligand interface during photoswitching.

A significant breakthrough in optical control was made by incorporating a catalyst directly within the organic monolayer, greatly expanding the range of transformations that can be light-regulated at the NP surface. This versatile approach enables photoswitchable control over virtually any catalyst or reaction compatible with the monolayer design. Sashuk and his team demonstrated this strategy by integrating an AB photoswitch with a proline organocatalyst, placing the photoswitch between the active center and the nanogold surface.^103^ In the *trans* configuration, the proline unit was exposed to the solution and remained catalytically active in an aldol condensation reaction. Upon isomerization to the *cis* form, the catalyst was buried within the monolayer, leading to reduced activity. In a subsequent study, the same group employed sterically demanding end groups, including a bulky ruthenium metathesis initiator, to completely block the active sites in the *cis* state—effectively shutting down a model olefin metathesis reaction (Figure 4IVb).^88^ Other than catalytic activity and selectivity, similar stimuli-responsive mechanisms can be used to control many other functions and behaviors associated with SAM@NPs, which are explored in Section 4 and 5.

### 2.3 Theoretical Descriptions of the Influence of Confinement within Catalytic On-NP Monolayers

As we have seen in the preceding sections, confining reactive or catalytic ligands to a mixed, on-NP SAMs can have two important consequences: (1) at higher concentrations, the proximity of these ligands can result in their cooperative interactions; and (2) if the reactive ligands are shorter than the surrounding, inert ones, the latter “gate” the access to the former and can modulate the selectivity on the substrates that reach these centers. Each case is interesting in terms of theoretical models aimed at understanding reaction kinetics.

Starting with case (1), one may consider a generic reaction A + B → AB catalyzed by some catalytic centers, X, distributed within the on-particle mixed monolayer. With a preponderance of inert “spacer” ligands, these X centers are distant, and it is reasonable to assume that the reaction rate (*R*) scales simply with their concentration (*C*_X_), *R*(*t*) = d*C*_AB_/d*t* = *C*_X_ *f*(*C*_A_, *C*_B_), where *f* is some function of reactants’ concentrations *C*_A_, *C*_B_. However, when concentration of X increases, these centers can become proximal, opening the possibility of cooperative catalysis involving X–X pairs, with the expression for rate becoming *R*(*t*) = d*C*_AB_/d*t* = *C*_X*–*X_ *f*(*C*_A_, *C*_B_). Mathematically, the concentration of the X–X pairs can be expressed by multiplying the concentration of *C*_X_ (number of X centers per volume of reaction mixture) by some probability, p_**_, that a second center X be present in its close proximity, *C*_X–X_ = *C*_X_ p_**_. In classical, solution models, it is assumed that X species are distributed uniformly in the volume of the reaction mixture, such that the probability p_**_ is proportional to *C*_X_, resulting in the well-known reaction rate proportional to *C*_X_^2^. This relationship changes dramatically when reactive sites/catalytic centers are confined within the on-NP SAM—uniquely, it is then possible to control p_**_ independently of *C*_X_, by diluting on-NP monolayers with inert “spacer” ligands (i.e., by modulating the on-particle fraction χ of the X centers while keeping the total concentration, *C*_X_, of these centers in solution constant, by adjusting NP concentration). As shown by the Grzybowski group in ref 104, a kinetics model can then be formulated by considering the surface attachment points of X-type ligands and, accounting for ligand conformational flexibility, the probability distributions *P*(**r**) of the reactive/catalytically active end groups, X (Figure 5A).

**Figure 5:**
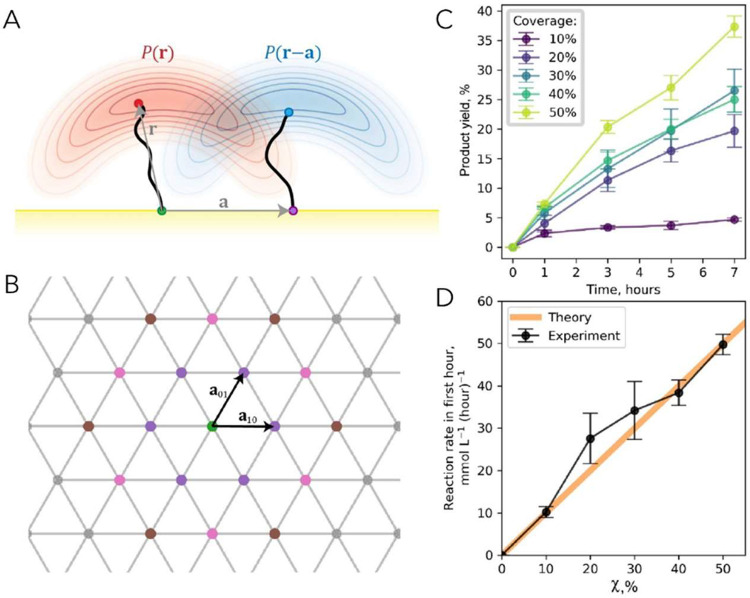
On-NP confinement and cooperativity. (A) Side view of two ligands (*black* curves) attached to the gold surface (*yellow*) at *green* and *purple* points separated by vector **a**. When flexible chains of the ligands are terminated in reactive/catalytically active groups X (*red* and *blue* points), the positions of these groups are defined by probability densities *P*(**r**) and *P*(**r**-**a**), shown by *red* and *blue* clouds, respectively. (B) Hexagonal lattice of ligand attachment points on the Au surface. Primitive lattice vectors **a**_01_ and **a**_10_ are shown. The main-text formula for p_**_ is derived by considering lattice points in the vicinity of the central (*green*) ligand and separated from it by |**a**_01_| (*violet* circles), |**a**_11_| (*pink* circles), and |**a**_02_| (*brown* circles). (C, D) If a particular reaction requires cooperativity between two catalytic centers X, then its rate is supposed to scale linearly with the on-particle fraction χ of the X centers (while keeping the total concentration of these centers in solution constant, by adjusting NP concentration). The data here are for the cycloaddition reaction of an azide with an acetylene, catalyzed by a NP-bound Cu catalyst, and confirm that product formation requires cooperativity between two X = Cu­(I) centers. Reprinted with permission from ref 104. Copyright 2022 American Chemical Society.

On a AuNP surface, the ligands are anchored to a hexagonal lattice of Au atoms with nearby lattice points separated by a translation vector **a** (Figure 5B). For two X-type ligands anchored at nearby lattice nodes, the probability that these two X centers are sufficiently close to each other to form an active X–X pair is a convolution (*) of two probability distributions: *P*(**r**)*P(**r**-**a**). Considering two lattice vectors, **a**_01_ and **a**_10_, and limiting the possible cooperative interactions to two “hexagonal belts” around point (0,0) (Figure 5B), it can then be shown that p_**_ = 1–(1–*χP*_01_)^6^ (1–*χP*_11_)^6^ (1–*χP*_02_)^6^ ≈ 6χ­(*P*_01_+*P*_11_+*P*_02_) where *P*_ij_ = *P*(**r**)*P(**r**-**a**_ij_) are convolutions between ligands tethered at different relative lattice points. The crux of this analysis is that—if there is indeed any cooperative effect of two proximal X sites—the reaction rates at some fixed time *t*_1_ and constant *C*_X_, *C*_A_, *C*_B_ should increase linearly with χ, *R*(*t* = *t*_1_) ∝ χ up to a saturation point. In ref 104, this scaling law was applied to provide an independent proof that the Nobel Prize-winning “click” reaction (i.e., copper–catalyzed azide–alkyne cycloaddition) requires cooperativity between two proximal X = Cu­(I) centers (Figure 5C,D). This cooperativity has long been debated and studied by advanced methods, including kinetic and isotope studies as well as synthesis of suitable complex mimics (for source references, see ref 104). This result highlights that catalytic SAM@NPs not only provide an artificial platform for reproducing enzyme-like catalytic behaviors but can also be a useful scaffold for probing mechanistic aspects of abiotic catalytic processes.

Of course, scaling formulas like the one derived above cannot address more nuanced proximity effects, in which probability distributions *P*_ij_ cannot be treated as constants—an example being when phase separation occurs among ligands in mixed SAMs; in such situations, one must describe on-particle microenvironments in atomistic detail. In this genre, Szleifer and Grzybowski^28^ considered how the AuNP radius (and other parameters) affects the p*K*_a_ of COOH end groups on alkanethiolate ligands; in this problem, the equilibria between protonated and deprotonated forms depend on the distances between and SAM-confined orientations of proximal acid sites (roughly speaking, it is harder to abstract a proton from COOH if another COOH in its vicinity is already deprotonated). Addressing this problem required consideration of the balance between electrostatic, van der Waals, steric, and packing interactions. To account for these effects and reproduce the experimentally observed trends, it was necessary to calculate and minimize the free energy of the system (within semi grand-potential formalism), explicitly including the coupling between the deprotonation equilibrium and all the relevant interactions. This type of analysis would be expected to inform the behavior of any SAM@NP catalytic process that involves the generation of surface-bound charge as an intermediate or transition state.

A conceptually distinct consequence of on-particle confinement is defined by situation (2) whereby ligands presenting reactive groups X and effecting some A → B transformation are shorter than the surrounding, nonreactive/inert ligands acting as access-controlling “gates”. In the simplest situation, the inert ligands may simply be long aliphatic chains that render the confined environment around X-sites lipophilic and potentially distinct to the bulk solvent environment. Under such circumstances, the concentration of substrates in solution may be different than that in the lipophilic pockets within the mixed SAM. This effect can be captured by considering partition-like coefficient *P*_monolayer/solution_^A^ = *C*_monolayer_^A^/*C*_solution_^A^ approximating the Gibbs free energy difference associated with entering the monolayer from bulk solution. This formalism treats the monolayer as a phase (in a thermodynamic sense) and in ref 79 was used to derive the linear relationship between *P*_monolayer/solution_^A^ and the corresponding and well-known octanol–water partition coefficient for the same species A, *P*_octanol/water_^A^. This, in turn, allowed linking the free energy of substrate A reaching sites X to this substrate’s lipophilicity, log *K* ^A^ = −Δ*G* ^A^/*k*_B_ *T* = const + β·log *P*_o/w_^A^. Using this expression, it was then possible to quantify the experimental selectivities should be with which different substrates *A*_i_ (characterized by different lipophilicities) entered the monolayer and underwent conversion at the X sites.

Finally, gating ligands that control access to the reactive X-sites may be terminated with groups that engage in specific intermolecular interactions with a substrate. For instance, charged end groups can control access of charged substrates (Figure 4Ia) or even orientation of substrates presenting nonsymmetric charge distribution (Figure 4Ib). In the latter case, ref 83 considered charged substrates, which upon entering the monolayer, had the charge center positioned at either radius *r*_1_ (measured from NP center) or *r*_2_. Then, the preference for one orientation over the other and, hence, the regioselectivity observed in the reaction catalyzed by site X, could be quantified by the difference in electrostatic energies, *selectivity* ∝ exp (−*q*(φ­(*r*_1_)−φ­(*r*_2_)) in which φ­(*r*) is the electric potential of the charged NP. While the physics of this approach can translate into more complicated substrates than those considered in ref 83, the approximations involved (e.g., presence of only two relevant substrate orientations) will likely be insufficient, calling for more advanced theoretical treatments or atomistic level simulations by MD.

Generally speaking, a further limitation to theoretical treatment is that—in common with systems-level analysis of any complex system—the effect of confinement on a single property (such as reaction rate or solvation, for instance) can be influenced by multiple interconnected factors operating across different length scales within the NP “system”,^105^ factors which are difficult to disentangle and properly capture in a physical model.^29^

## 3 Molecular Recognition and Sensing

### 3.1 Molecular and Biomolecular Recognition Using SAM@NPs

Ligand monolayers coating AuNPs exhibit several characteristics relevant to molecular recognition.^106,107^ Depending on the molecular structure of the ligands, there are several possible modes of interaction of the monolayer with molecular or macromolecular guests (Figure 6A), some of which were discussed in Section 2: (i) divergent multivalent interaction with the ligand end groups, (ii) convergent, tweezer-like, interaction with the ligand end groups, (iii) hydrophobic partitioning within the inner shell (only in water), and even (iv) convergent, cleft-like, multiple interactions within the monolayer.

**Figure 6:**
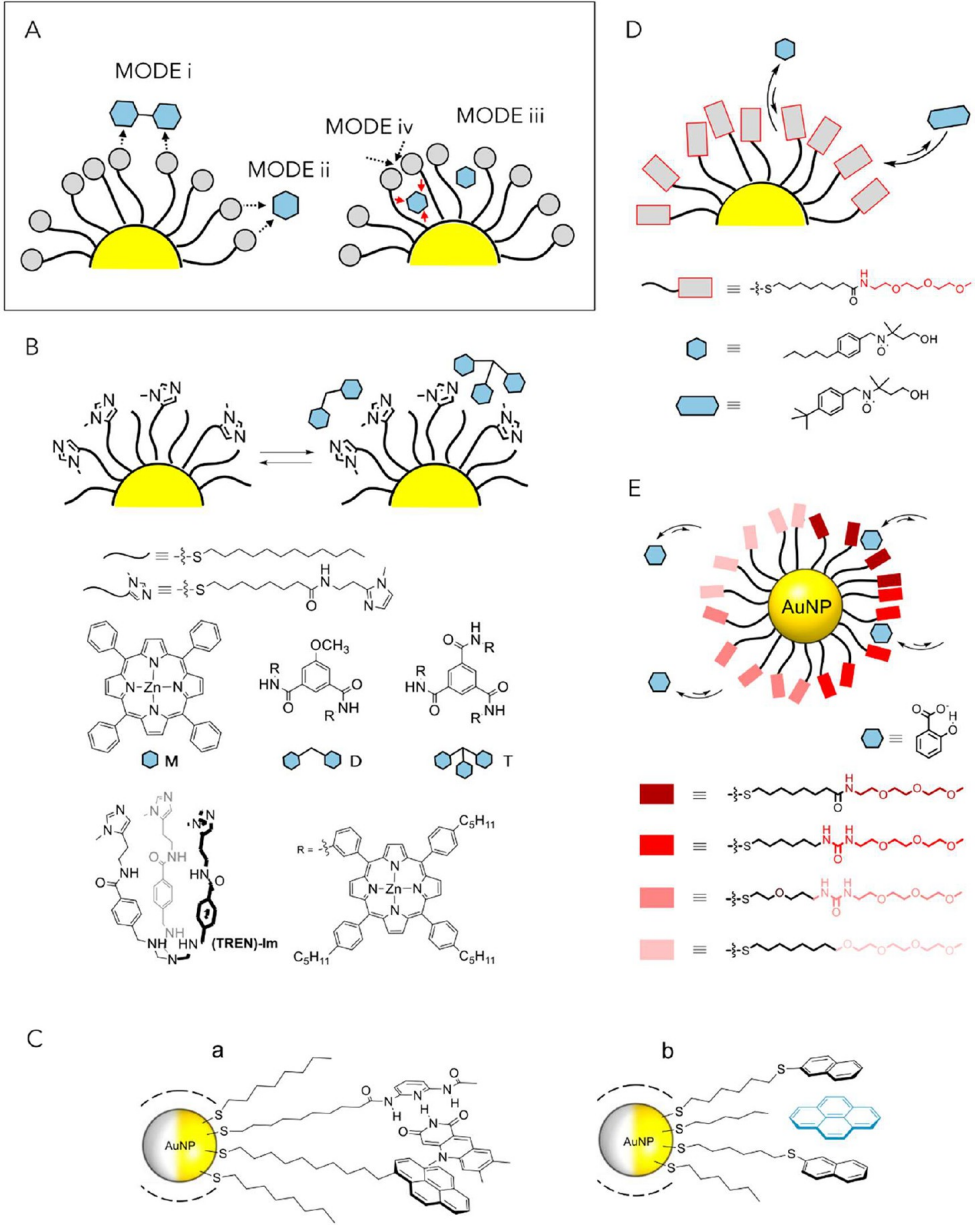
(A) Possible binding modes for SAM@NP interacting with recognition partners. *Mode i*: divergent multivalent binding; *mode ii*: tweezer-like binding; *mode iii*: hydrophobic partitioning; *mode iv*, cleft-like binding. (B) Multivalent interactions between *N*-methyl-imidazole and porphyrin arrays.^108^ (C) Tweezer-like binding mode in ligand protected NPs; (a) multipoint flavin recognition,^109^ and (b) π-stacking based recognition of pyrene (adapted with permission from ref 110. Copyright 2011 Wiley-VCH GmbH). (D) Bulkiness-based partitioning of organic radical within SAM@NPs.^111,112^ (E) Differential molecular recognition of salicylate by SAM@NPs coated by neutral-water solubilizing ligand.^113^

Compared to other multivalent binders, SAM@NPs bring about relevant advantages (*mode i*—Figure 6A). First, the grafting of the ligands to the Au surface is expected to compensate for most of the entropy loss caused by multivalent binding, while allowing enough flexibility to accommodate with the requirements of the binding partner. Second, the self-organized nature of SAM strongly alleviates the synthetic costs.^108^ In a work dated 2003, Pasquato and Scrimin measured the affinity between SAM@NPs with a mixed monolayer of alkyl and *N*-methylimidazole functionalized thiols and Zn^2+^-porphyrin multimers synthesized by the group of Hunter (Figure 6B).^108^ They found that the affinities of a Zn^2+^-porphyrin dimer (D) and trimer (T) for the SAM@NPs were respectively 2 and 3 orders of magnitude larger than that of the corresponding monomer (M). Even though increasing the number of interactions led to a less than additive affinity increase, the effective molarity calculated for the intracomplex binding of the second and the third porphyrin unit (1 mM) was substantially larger than that previously measured for the flexible tripodal ligand tris­(2-aminoethyl)­amine (TREN)-Im (0.05 mM).^114^ This observation confirmed the effectiveness of the preorganization obtained by the grafting of the binding sites to the NP.

Multivalent receptors are present, *par excellence*, in biological systems, mediating, for example, cell-surface recognition in the immune system and in viral infections. As a consequence, the multivalent nature of SAM@NPs makes them promising candidates as synthetic binders for biological systems. Surprisingly, this topic was scarcely investigated until recently. At the end of the 2000′s, it was demonstrated that functionalized AuNPs were effective virustatic agents against HIV and Herpes fusion with cells.^115,116^ In both the cases, the presence of multiple ligand copies on the surface of the NPs resulted in a strong increase of the affinity and specificity toward different viruses. It was however only in 2018 that this approach received a wider attention thanks to the work of Stellacci, who demonstrated how tuning of structure of the NP coating ligands by the introduction of a lipophilic portion converted their activity from virustatic to virucidal.^117^

Carbohydrate-functionalized NPs, or glyconanoparticles, where multiple saccharide copies are displayed at the NP surface, have been widely explored to model carbohydrate–carbohydrate and carbohydrate–protein interactions in cell adhesion processes and to quantify their binding strength. These interactions are inherently weak, but their multivalency compensates for this limitation, mimicking the behavior of biologically relevant polysaccharides. Notably, the use of SAM@NPs to study carbohydrate–carbohydrate interactions was pioneered by Penadés and co-workers, who first employed AuNPs functionalized with the trisaccharide Lewis X (Le^X^) to investigate the calcium-dependent, reversible self-aggregation of Le^X^.^118,119^ The natural application of these systems lies in the targeting of cell surfaces, offering promising prospects for therapeutic and diagnostic applications.^120–122^

Advantages of the multivalent binding ability of SAM@NPs are of course strongly emphasized when the coating ligands feature charged end groups. In this case, host–guest association can rely on the long-range, nondirectional and additive nature of ion pairing interactions. This concept was early on demonstrated by Rotello who, in 2004, investigated the interaction of trimethylammonium functionalized AuNPs with peptides containing four aspartate residues, showing strong binding (micromolar affinities) and significant stabilization of the peptide secondary structure.^123^ Research on ammonium-functionalized SAM@NPs continued in the Rotello group, with a focus on finely tuning the hydrophobicity of the end groups. They demonstrated that cationic SAM@NPs are highly effective in binding polyanionic species such as polymers, proteins, and even cells. These systems were eventually applied to the differential sensing of biologically relevant entities, as further discussed below in this section (e.g., Figure 9).^124,125^ Later on, Prins investigated the interaction between positively charged SAM@NPs and polyanionic molecular targets such as nucleotide di- and triphosphates, demonstrating that nanomolar affinities can be reached,^126^ and establishing another design motif that has formed the basis of diverse functional SAM@NPs (e.g., Figures 10–12).

The possibility to exploit the spatial proximity of the ligand end groups to obtain convergent, tweezer like, multiple interactions with the guest (*mode ii*—Figure 6A) had been recognized even earlier by Rotello. In a pioneering investigation dated 2000, his group demonstrated that mixed monolayer AuNPs featuring pyrene and 2,6-diamidopyridine moieties were able to recognize flavin in chloroform (Figure 6C­(a)).^109^ The multipoint convergent interactions with the guest, resulting from hydrogen-bonding from the diamidopyridine and π-stacking from the pyrene unit, led, for the optimized SAM@NPs composition, to a 4-fold enhanced affinity for the substrate with respect to the SAM@NPs featuring only the diamidopyridine moiety. A similar binding mode was implicit in the substrate recognition brought about by the SAM@NPs coated with ligands featuring Zn^2+^ complexes of 1,4,7-TACN,^54^ which were indeed capable of accelerating the cleavage of phosphate diesters with Michealis-Menten kinetic profiles (see Section 2), implying the tweezer-like bimetallic molecular recognition of the substrate.

Tweezer-like binding modes have been explored over the years by other groups. In 2011, Stellacci reported that films made by mixed monolayer AuNPs coated with a naphthalene and alkyl ligands were capable of binding polycyclic aromatic compounds from ethanol by exploiting sandwich π-stacking interactions (Figure 6C­(b)).^110^ Quite remarkably, SAM@NPs coated with the naphthalene ligand only were unable to bind the same substrates. This suggested that the shorter alkyl thiol played the fundamental role of decreasing the surface density of the naphthalene residues, preventing their self-aggregation and inducing the formation of tweezer-like binding sites. A few years later, Mancin investigated the ability of coating with ligands featuring a 18-crown-6 ether end group to bind protonated primary amines in methanol.^127^ Here, the effect of grafting the interacting groups to the AuNPs was similar. Affinities toward protonated monoamines were 10-fold smaller compared to the free crown ether, while an unprecedented selectivity for primary amines versus amino acids was found. This behavior was also ascribed to the steric crowding of the binding units in the monolayer. On the other hand, diamines such as cadaverine and putrescine were bound with 4–6 fold higher affinities, compared to monoamines, as the result of a two-point interaction. In a related example, Kubik demonstrated that mixed monolayer AuNPs coated with three different thiols, featuring a 18-crown-6 ether, a trimethylammonium and a phenyl moiety, were able to bind dipeptides in water.^128^ As in the system studied by Stellacci (Figure 6C­(b)), it was found that the role of one of the coating thiols, namely the phenyl one, was to dilute the other two favoring the formation of more suitable crown ether/ammonium binding motifs. It is relevant to note that in this case, as in the Stellacci one, the dilution of the crown ether moieties in the monolayer was necessary to recover their molecular recognition capabilities.

The possibility of hydrophobic species to partition in the inner portion of the monolayer, acting as a low polarity pseudophase in an otherwise aqueous environment, has been proposed in several cases for SAM@NPs (*mode iii*—Figure 6A) and theoretically interpreted in specific contexts (see Section 2 and ref 79 therein). In many examples, hydrophobic interactions were found to complement end group multivalent or tweezer-like interactions resulting in an increased affinity for the target guest.^129–131^ Detailed investigations on hydrophobic partitioning have been performed by Pasquato, Lucarini and co-workers over the years, in an attempt to disentangle the mutual dependence of NP curvature, hydrophobic interactions, monolayer structure and topological features of the monolayer surface. The binding of a series of neutral radical probes to SAM@NPs coated with noncharged, water-solubilizing ((oligo)­ethylene glycol end group) ligands was studied by electron spin resonance (ESR) spectroscopy (Figure 6D).^111,112^ As expected, an increase of the guest affinity for the NP when the substrate lipophilicity increased was observed. More interestingly, the affinity for the substrate decreased when the NP size was increased. This latter effect was attributed to the denser packing of the coating ligands induced by the reduced curvature of the metal core, resulting in greater energetic costs for the reorganization of the ligands needed to accommodate the guest within the monolayer.

Amphiphilic fluorinated monolayers were found to be particularly effective in creating high-affinity endoreceptors for small hydrophobic molecules. They could be used either alone, in homoligand monolayers, or as patches within mixed monolayers.^132,133^ This exceptional affinity arises from their high hydrophobicity but must also be complemented by sufficient flexibility in the ligand chain. Flexible ligands with fluorinated subunits in a distal position provide greater conformational freedom, allowing for better adaptation and fitting during binding. In contrast, ligands with rigid fluorinated segments positioned closer to the Au core show reduced flexibility, leading to less effective binding. As a result, the equilibrium partition constant of a nitroxide probe (used as a guest model) decreases by an order of magnitude for rigid chains.^134^

Similar conclusions on the role of ligand conformational mobility on molecular recognition with SAM@NPs were drawn by Mancin, Rastrelli and De Vivo. They investigated the binding of sodium salicylate to a series of AuNPs coated with noncharged, water solubilizing ligands (Figure 6E).^113^ Counterintuitively, the absence of the amide/urea linking group between the alkyl and (oligo)­ethylene glycol portion of the ligand, on AuNPs with the same size, was detrimental for the guest binding. Even more surprisingly, the presence of an oxygen atom in position 3 of the alkyl chain (close to the Au surface) also strongly reduced the affinity for the guest.^135^ The reasons for such behaviors were provided by extensive MD simulations, which indicated that both ligand bundling due to hydrophobic interactions, or increased monolayer packing due to the heteroatom ability to induce *gauche* conformations, reduced the monolayer mobility and increased the energetic cost of pocket opening (*mode iv*—Figure 6A).^136^ In many cases described in the literature, including some of those discussed here, the molecular recognition mode of SAM@NPs (when not limited to multivalent end groups or tweezer like interactions) closely resembles that of cavitands^137^ featuring a hydrophobic interior and a functionalized portal. This evidence points to the possibility to design the ligand shell in such a way as to obtain binding pockets similar to those present in proteins or enzymes.

In many amphiphilic ligands described above, the hydrophilic component is provided by an (oligo)­ethylene glycol segment. However, smaller polar moieties can also ensure water solubility while introducing the possibility of forming hydrogen bonds with water, other terminal groups, and other species in solution. This can lead to different monolayer structures, ultimately affecting accessibility, solvation, and hosting capabilities.^138^ Figure 7A outlines NP features influencing structural variation in SAMs. These key factors do not operate independently; rather, the resulting local environments arise from their combined effects. This interplay makes anticipating the resulting organization a complex task. However, recent computational studies have begun to reveal correlations between molecular structure and monolayer morphology that can be translated into general design guidelines. In particular, small charged end groups, such as sulfonate groups, promote the formation of long-living ligand clusters, where interligand electrostatic repulsion is balanced by water bridges, hydrogen bonding and counterion condensation; under such conditions, guests accommodate between ligand bundles and monolayer hydration is poor inside the bundles (Figure 7B). In contrast, quaternary ammonium groups, with lower charge density and higher bulkiness, result in a uniform, radial ligand distribution with the guests placed inside the monolayer (Figure 7B). Similarly, zwitterionic end groups (*e.g*., −PO^4–^(CH_2_)_2_N^+^(CH_3_)_3_) lead to a radial arrangement, stabilized by hydrogen bonding networks.^138^ In addition to bulkiness and charge of the end group, thick monolayers (with chain length typically longer than 12 C atoms) promote multiple distinct microenvironments of varying polarity inside the monolayer for small guest molecules. For instance, when ligand bundling occurs, guest molecules can explore two positions at the boundaries between bundles or laying down between them (Figure 7C). Generating surface domains (*e.g*., patches) by mixing phase-separating ligands further expands the range of environments available to an incoming molecule at a SAM@NP.^139^

**Figure 7:**
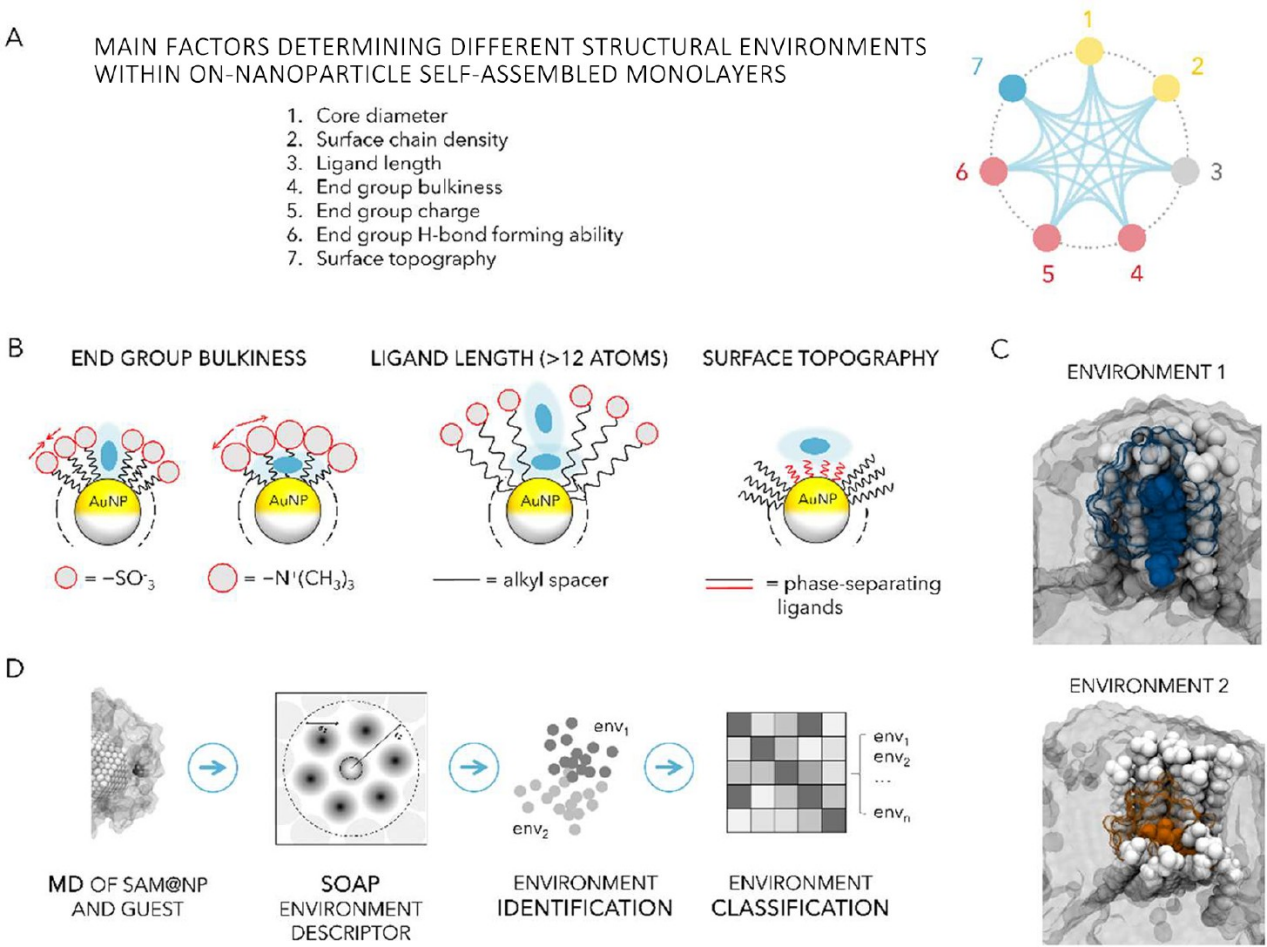
(A and B) Main factors influencing monolayer organization and formation of different local hosting environments in *mode iii* binding (see Figure 6A for categorization of binding modes). Core (yellow soild circle), ligand (gray soild circle), end group (red soild circle) and monolayer composition (blue soild circle) -related factors. (C) Example of two distinct binding poses and their associated local environments within a SAM@NP featuring a thick C-16 shell. The guest is colored in blue/orange based on the environment. Reprinted with permission from ref 139. Copyright 2022 American Chemical Society. (D) Local environments can be identified through data-driven analysis of MD trajectories of SAM@NP interacting with the guest, exploiting unsupervised machine-learning techniques in combination with the smooth overlap of atomic positions (SOAP) structural descriptor. Reprinted with permission from ref 139. Copyright 2022 American Chemical Society.

Posocco and Pasquato have recently demonstrated that combining unsupervised machine-learning approaches with MD simulations enables the identification, comparison, and classification in terms of similarity of local environments experienced by small hydrophobic probes interacting with SAM@NPs, without need of any *a priori* knowledge or assumptions^139^ (Figure 7D).

This study also demonstrates the huge and crucial potential of computer simulations to assist the rational design of a SAM@NP supramolecular host. Indeed, simulations not only provide additional insights into the binding modes and sites of experimental systems,^140^ as discussed so far, but also now allow virtual screening of SAM@NP structural mutations or libraries to tune the nanosystem affinity for the target guest *in silico*. In an early example, Mancin and De Vivo demonstrated that atomistic MD simulations on a small library of SAM@NPs bearing amide, urethane and urea moieties, present in the central part of the alkyl thiolate structure, could be used to rank their affinity for salicylate, leading to the selection of a candidate with 10-fold affinity higher compared to early candidates.^135^ Very recently, the same authors reported on a method for the fast computational screening of a 100-mer library of AuNPs coated with tripeptides, allowing the selection and experimental validation of the candidate with the highest affinity for the cancer biomarker 3-methoxythreonine (3-MT) (Figure 8).^141^

**Figure 8:**
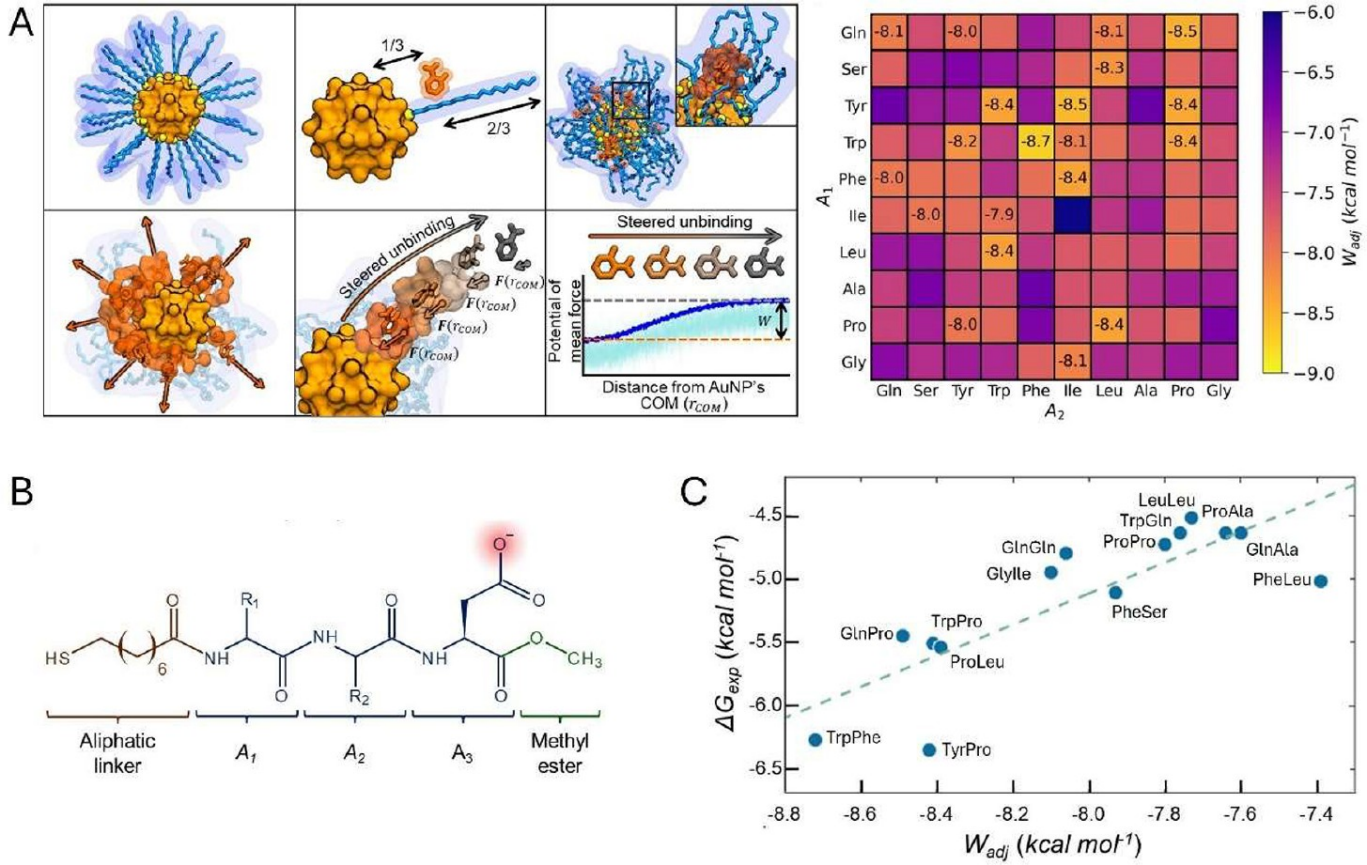
(A) Computational affinity assessment and scoring of a library of tripeptide-based SAM@NPs toward 3-MT. Affinity is computed by pulling out the analytes from the monolayer with steered MD simulations and then calculating the adjusted binding score (*W*_*adj*_). (B) Library of thiols screened; the outermost amino acid (A_3_) is always Asp. (C) Correlation between the computational scores (*W*_adj_) and the experimental binding free energies *(*Δ*G*_exp_), for 14 tested SAM@NP/3-MT systems. All figures are reprinted with permission from ref 141. Copyright 2025 Royal Society of Chemistry.

### 3.2 Sensing in Solution Using SAM@NPs

One of the most typical applications of molecular recognition is sensing.^142^ Chemosensors are chemical systems where the formation of a host–guest complex induces the variation of some measurable property of the sample. Such variation, in turn, allows the detection and quantification of the guest. The binding of molecules to SAM@NPs normally produces relevant modifications of some of their properties. These include (weak) coupling effects such as the increase in the intensity of Raman signals or the modification of excited state lifetimes (luminescence quenching or enhancement), as well as dynamic effects such as decrease in the conformational mobility, the diffusion rate or the tumbling rate, any of which can be exploited as signal-generating properties. Among these phenomena, the ability of AuNPs to quench the fluorescence emission of dyes located in their proximity is the property most often exploited for sensing.^143,144^ This effect works over relatively long ranges. For these reasons, it is particularly suited for the development of IDA (Indicator Displacement Assay) sensing schemes.^145^ IDA is based on the displacement of an indicator molecule from the host upon analyte binding. The change of chemical environment experienced by the indicator leads to a change of its properties and to the consequent generation of the read-out signal. In the case of SAM@NPs, the analyte-induced displacement of a dye bound to the monolayer intrinsically results in the restoration of its fluorescence emission.

The IDA approach, combined with multivalent guest recognition, has been pioneered and then extensively used by Rotello’s group to develop pattern-based sensing systems for proteins, cells and bacteria based on libraries of cationic SAM@NPs and anionic dyes or fluorescent polymers. A prototype sensor array was developed using six structurally distinct cationic SAM@NPs and an anionic poly­(*p*-phenyleneethynylene) polymer.^146^ As shown in Figure 9A, electrostatic complexation of SAM@NPs and polymer results in fluorescence quenching of the polymer (fluorescence “OFF”) through energy transfer. Addition of protein analytes then disrupts the quenched polymer-SAM@NPs complexes via competitive binding, causing fluorescence recovery of the polymer (fluorescence “ON”). Protein-NP interactions are selective, leading to a fingerprint fluorescence response pattern for individual proteins (Figure 9B). By employing the same principle, a SAM@NP-(green fluorescent protein) construct was used to detect and identify proteins at 500 nM in undiluted human serum (∼1 mM overall protein concentration).^147^

**Figure 9:**
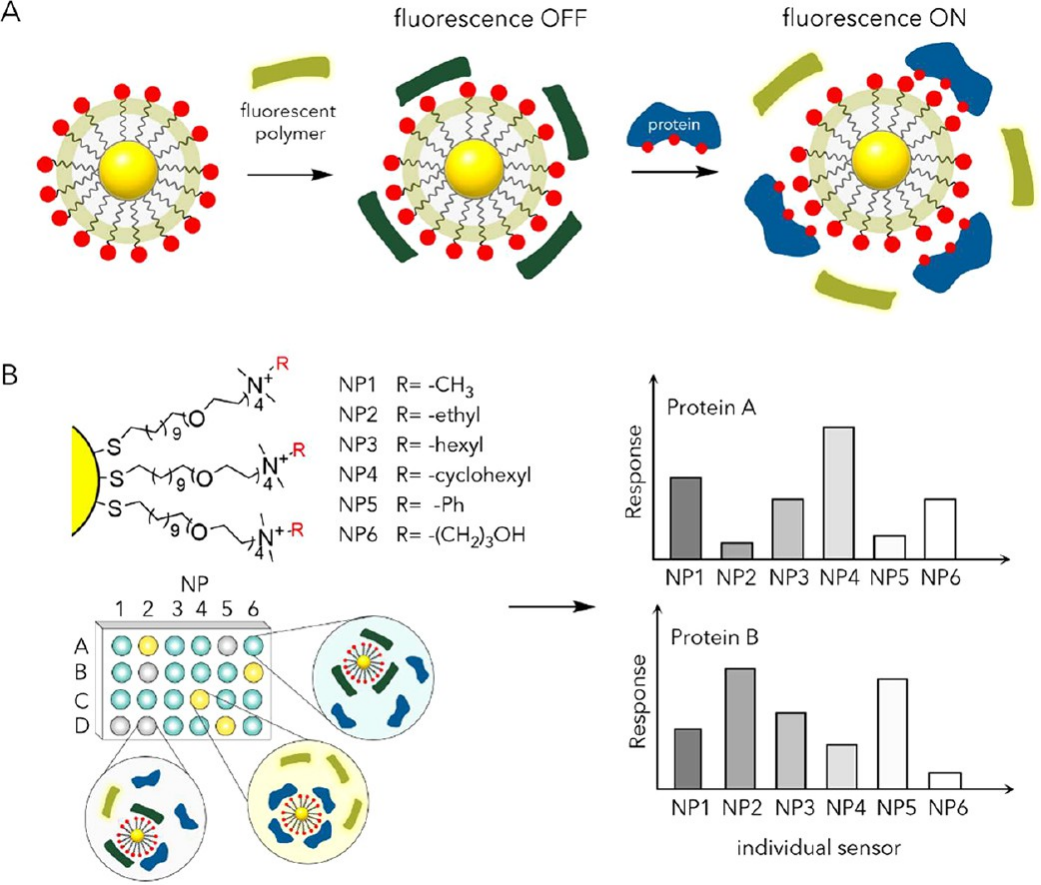
(A) Pictorial representation of protein sensing mechanism via competitive binding between the protein and the quenched polymer-SAM@NP complex.^146^ (B) The SAM@NP end groups incorporate hydrophobic, aromatic, or hydrogen-bonding functionalities to modulate interactions with proteins. The combination of an array of sensors generates fingerprint response patterns for individual proteins.^146^

Prins and co-workers expanded the concept by employing SAM@NPs terminated with TACN-Zn^2+^ end groups (*i.e*., SAM@NP **1**•Zn^2+^, Figure 10A) to construct multivalent supramolecular structures in aqueous media, a research line that the group has explored over several years, building increasingly complex yet elegant systems. Initially, the authors demonstrated that SAM@NP **1**•Zn^2+^ (2 nm core size) can bind quantitatively up to 18 oligoanions (as nucleotide triphosphates or diaspartate-terminated peptides) per NP, encompassing phosphates and carboxylates at concentrations even below micromolar.^148^

**Figure 10:**
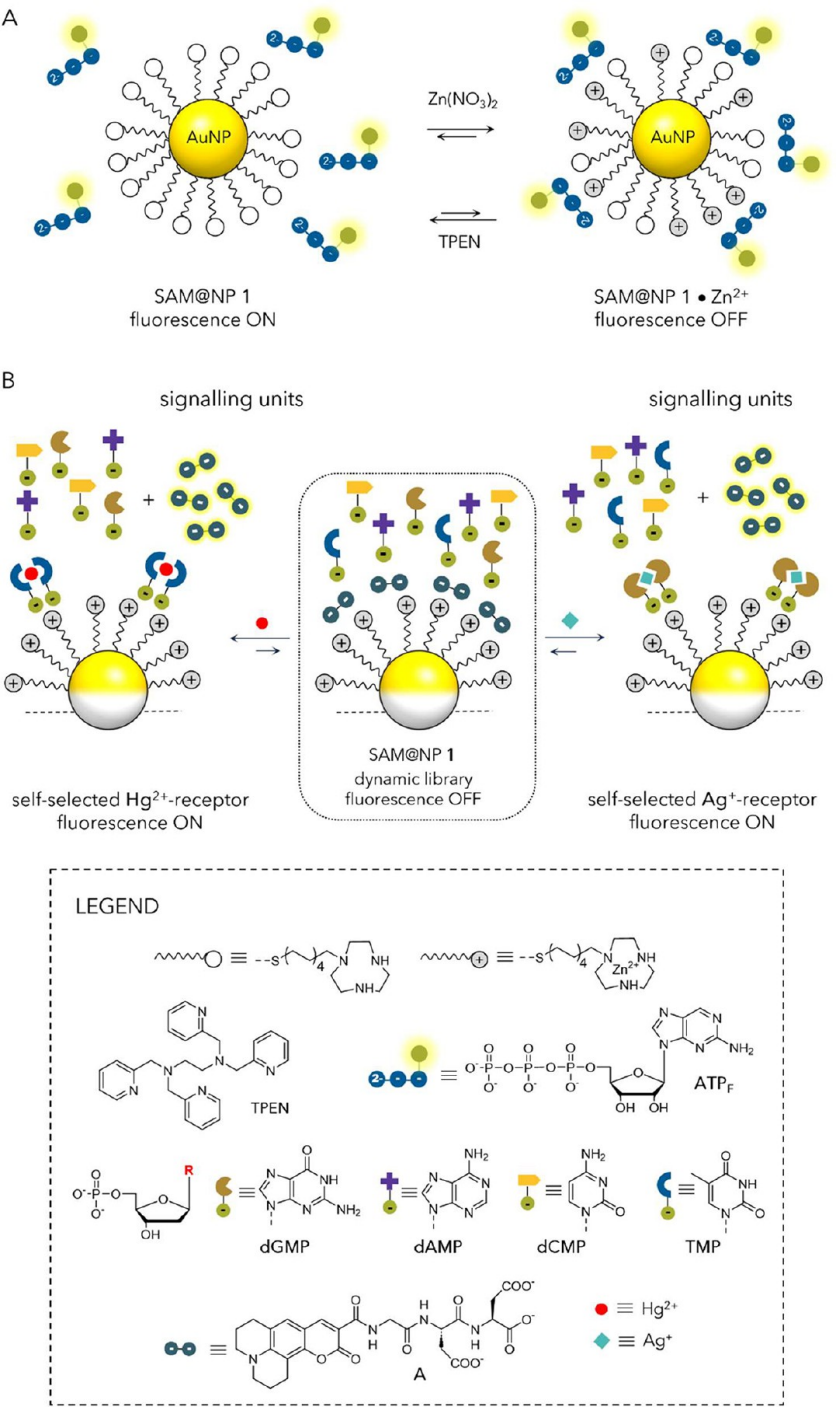
(A) Schematic representation of metal-mediated control over the valency of the TACN-SAM@NP-based supramolecular system developed by Prins and co-workers. Reprinted with permission from ref 149. Copyright 2012 American Chemical Society. (B) Schematic representation of metal-mediated self-selection: complex formation between two recognition units and a metal ion results in the formation of a ternary complex with a high affinity for SAM@NP **1**•Zn^2+^, resulting in the displacement of fluorescent probe A from the surface, activating its fluorescence. Reprinted with permission from ref 150. Copyright 2015 Royal Society of Chemistry.

To demonstrate that Zn^2+^ ions could act as a regulatory element of valency, responsible for the interaction with the oligoanions in the SAM@NP **1**•Zn^2+^ system, the authors^149^ added Zn­(NO_3_)_2_ to a solution containing SAM@NP **1** and 2-aminopurine riboside-5′-O-triphosphate (ATP_F_), a fluorescent analogue of ATP at pH 7.0 (Figure 10A). This addition resulted in the metalation of the TACN ligands and the subsequent capture of unbound ATP_F_ by the Zn^2+^ complexes at the SAM@NP surface. The formation of this complex was detected via fluorescence spectroscopy, utilizing the quenching ability of SAM@NPs. Subsequently, the removal of Zn^2+^ ions using *N*,*N*,*N′*,*N′*-tetrakis­(2-pyridylmethyl)­ethylenediamine (TPEN), a ligand with a higher affinity for Zn^2+^ ions than TACN, resulted in the release of ATP_F_ and full recovery of the initial fluorescence intensity, indicating complete reversibility of the process. The process is pH-dependent, with higher pH levels reducing surface binding and accelerating molecule release rates. The authors also demonstrated that analogous results, but with different kinetics, can be obtained by replacing Zn^2+^ ions with Cu^2+^ ions.

Interestingly, the SAM@NP **1**•Zn^2+^ system exhibited a higher affinity for phosphate probes as compared to carboxylate probes. This selectivity enabled the authors^151^ to achieve self-sorting of phosphate and carboxylate molecules on the surfaces of two SAM@NPs. The selective interactions between phosphate probes and SAM@NP **1**•Zn^2+^ system caused the carboxylate probes to relocate to a different type of NP, specifically SAM@NPs with quaternary ammonium end groups.

High local concentrations promoted by colocalization is useful for sensing: inspired by the selective binding of Hg^2+^ to the DNA nucleobase thymine (T) forming a [T•Hg^2+^•T] complex, Prins^152^ employed SAM@NP **1**•Zn^2+^ for the detection of Hg^2+^ ions at low nanomolar concentrations through a signal transduction pathway involving multivalent interactions (Figure 10B). The analyte (*i.e*., Hg^2+^ ions) induces the dimerization of low-charge ligands (*i.e*., thymine diphosphate, TDP), forming a high-charge species with high affinity for multivalent SAM@NP **1**•Zn^2+^. This complex displaces a quenched fluorescent reporter (*i.e*., probe A) from the SAM@NP **1**•Zn^2+^ surface, resulting in fluorescence activation. The authors^150^ also showed that the addition of Hg^2+^ or Ag^+^ ions to a dynamic system composed of SAM@NP **1**•Zn^2+^, and a mixture of four nucleotides (dGMP, dAMP, TMP, and dCMP) leads to the spontaneous self-selection of TMP or dGMP, respectively, on the monolayer surface. The capability to utilize dynamic combinatorial chemistry on SAM@NPs presents significant potential for developing highly complex multivalent receptor systems.

The same SAM@NP **1**•Zn^2+^ system was also able to bind several di- and tri- nucleotides with distinct affinities.^153^ The range of affinities was sufficient to achieve quantitative discrimination of all the nucleotides at low mM concentrations.

The systems described above generate signals through thermodynamically driven processes, where a molecular recognition event shifts the system into a more stable state, causing a measurable property change. The energy required for indicator dissociation is outweighed by the energy gained from receptor–analyte complex formation. Introducing a return mechanism would enable multiple signaling cycles and temporal control over signal intensity, allowing the system to mimic natural signaling pathways. Prins^154^ developed a strategy for transient signal generation in a self-assembled system. This approach includes control over signal intensity and decay rate, concentration-dependent activation of different pathways, and transient downregulation of catalytic activity (Figure 11). Similar to the previous examples, the fluorescence of probe A is quenched when bound to SAM@NP **1**•Zn^2+^, but its emission is restored upon displacement by ATP nucleotide. The authors demonstrated that this system can be used to generate a transient fluorescent signal by triggering the irreversible hydrolysis of ATP (*i.e*., fuel) into AMP and inorganic phosphate (Pi) using the enzyme potato apyrase. This process results in the reformation of the complex between probe A, which has higher affinity than AMP, and SAM@NP **1**•Zn^2+^, causing the fluorescence signal to disappear, thus demonstrating the transient nature of signal generation akin to natural systems (Figure 11A).

**Figure 11:**
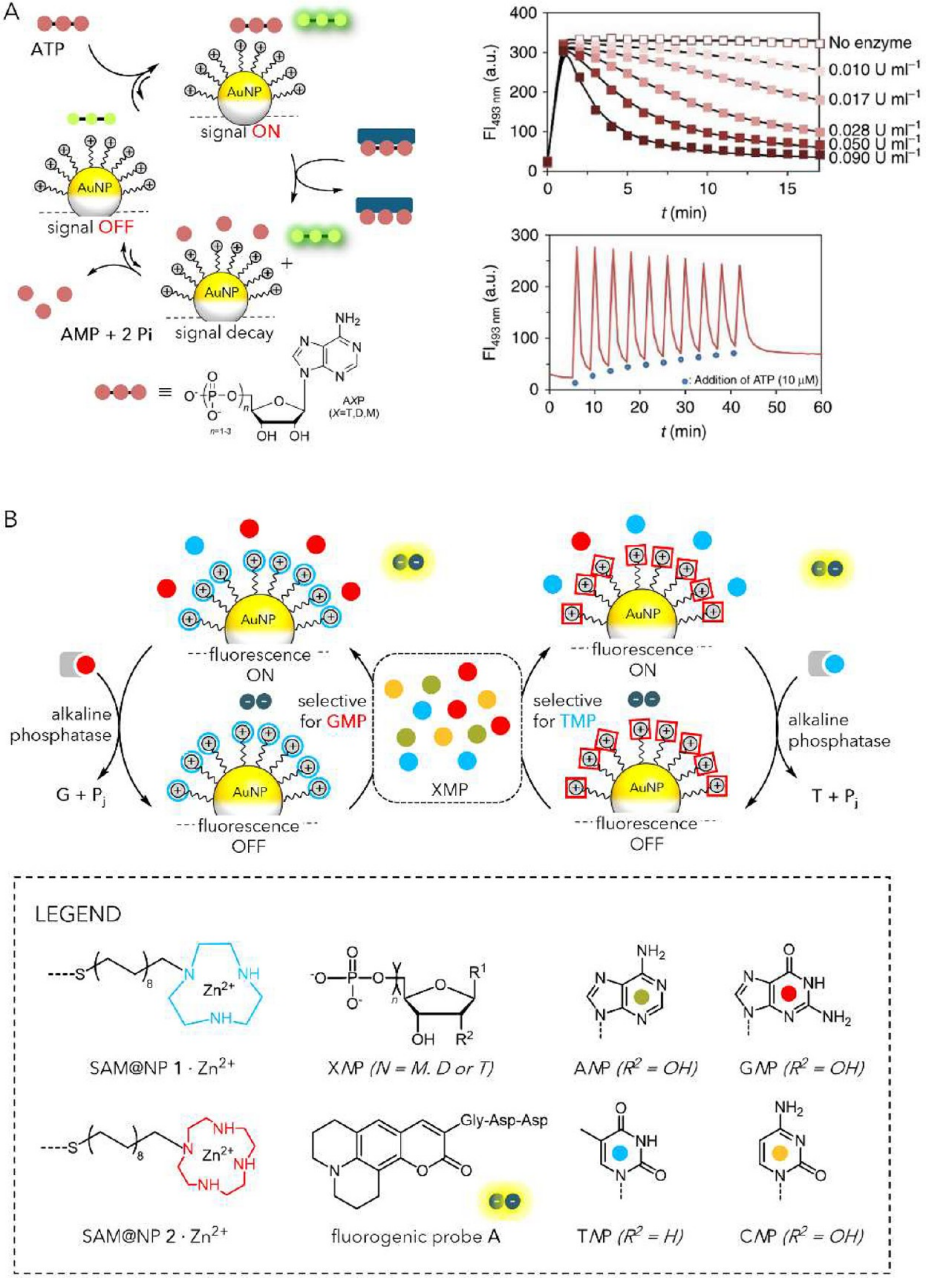
(A) Scheme of the system for transient signal generation fueled by ATP (left), intensity as a function of time on the addition of ATP to SAM@NP **1**·Zn^2+^ (right, top) and after repetitive additions of ATP in the presence of the enzyme (right, bottom). The enzyme is depicted in blue, and the fluorogenic probe - identical to that shown in Figure 10B—is shown in green. Reprinted with permission under a Creative Commons Attribution 4.0 International license from ref 154. Copyright 2015 Macmillan Publishers Limited. (B) Illustration of the selective interaction of GMP and TMP with the surfaces of SAM@NP **1**·Zn^2+^ and SAM@NP **2**·Zn^2+^, leading to the generation of a transient signal. Reprinted with permission from ref 155. Copyright 2018 Wiley-VCH GmbH.

As a follow-up, the same authors^155^ demonstrated that the selectivity toward the fuel can be tuned by modifying the end group of the SAM@NP (Figure 11B). They specifically investigated SAM@NP **1**•Zn^2+^ and a newly studied variant with a 1,4,7,10-tetraazacyclododecane (cyclen) end group (*i.e*., SAM@NP **2**•Zn^2+^). These systems exhibited selective binding to specific nucleotides (*e.g*., GMP for SAM@NP **1**•Zn^2+^and TMP for SAM@NP **2**•Zn^2+^). The study further explored the dynamic interactions in a mixture of both NP types, finding that the selectivity could shift based on the nucleotide concentration.^155^

As an alternative to conventional sensing protocols, NMR spectroscopy offers several different tools to convert SAM@NPs with molecular recognition abilities into chemosensors. The binding of a small molecule to a larger entity, and the inclusion in a molecularly crowded environment, such as the coating monolayer, strongly affects properties like diffusion rate, tumbling rate and conformational mobility. The direct result of such changes on the spectral features of the bound molecules is generally line broadening. This effect, *per se*, is poorly suitable for sensing application, since the modification or even disappearance of the single signal in a crowded spectrum is difficult to detect. Nonetheless, several NMR-based experiments demonstrate the ability to differentiate the species of interest according to their mobility. Another key advantage of NMR spectroscopy lies in its ability to provide direct structural information about the analyte. As a result, sensing does not depend on designing a monolayer that binds exclusively to a single species and, in principle, unknown analytes can be identified. In a DOSY experiment, the signals due to different components of a mixture are separated according to their diffusion coefficient. Akin to chromatography, differences in diffusion coefficients allow for the separation and identification of NMR signals from individual species (“chromatographic NMR”).

Mancin and Rastrelli demonstrated that SAM@NPs perform quite well as a stationary phase for chromatographic NMR since they allow different interaction modes while not perturbing the magnetic field homogeneity of the sample.^156^ Accordingly, SAM@NPs coated with alkylammonium, or alkyl sulfonate ligands differentiated mixtures of organic anions or cations (Figure 12A). This approach was simplified to the limit of using fast and sensitive monodimensional experiments (diffusion filters) in combination with high affinity SAM@NP receptors. In this case, the diffusion rate of the guest was nearly the same as the SAM@NPs. Hence, AuNPs coated with ligands terminated in Zn^2+^ complexes (SAM@NP **1**) were able to detect organic phosphates at 50 μM concentration, in the presence of 0.5 mM benzoate, with a 30 min long experiment.^157^

**Figure 12:**
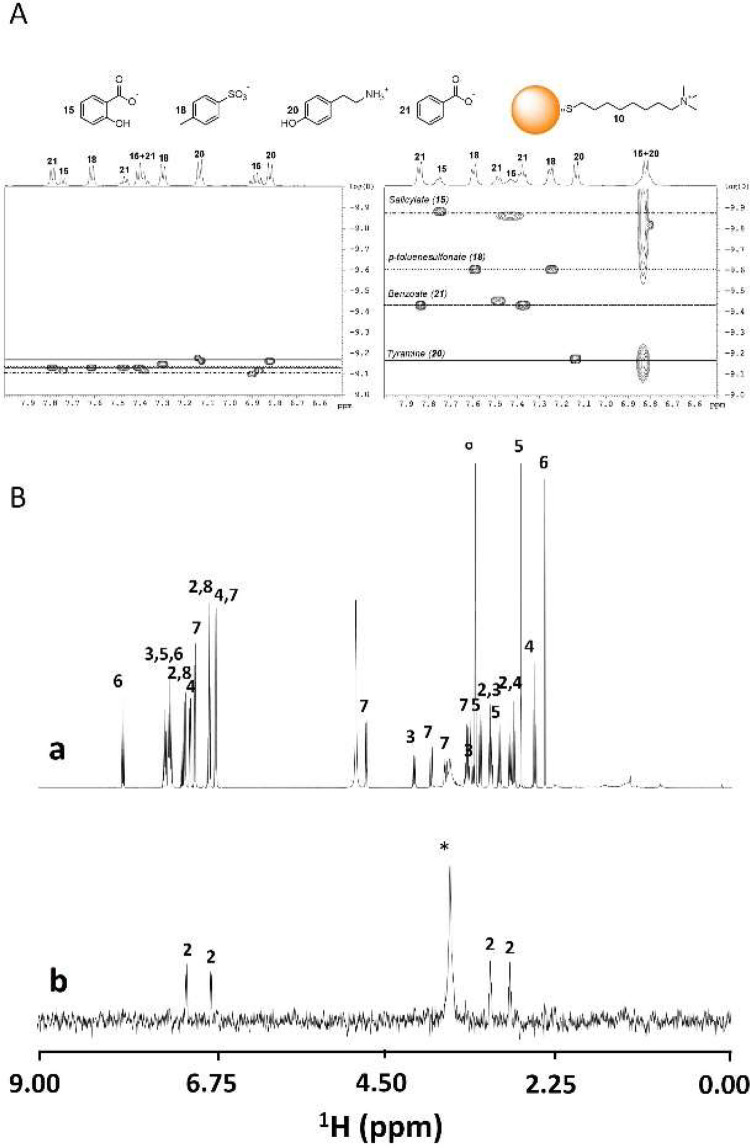
(A) ^1^H DOSY maps resulting from a mixture of sodium salicylate (**15**), potassium *p*-toluenesulfonate (**18**), sodium benzoate (**21**), and tyramine (**20**) in water in the absence (left) or in the presence (right) of AuNPs coated with ammonium ligands. Adapted with permission from ref 156. Copyright 2014 American Chemical Society. (B) ^1^H NMR spectra resulting from a mixture of tyramine (**2**), l-phenyalanine (**3**), phloretic acid (**4**), *N*-methylphenethyl amine (**5**), *p*-toluic acid (**6**), arbutin (**7**), and clorophenol (**8**) in water and in the presence of AuNPs coated with 18-crown-6 ligands recorded (a) with a standard ZG sequence, (b) with the NOE pumping-CPMGz sequence. Reprinted with permission from ref 127. Copyright 2015 American Chemical Society.

More sophisticated NMR detection protocols are based on the transfer of magnetization/saturation from the SAM@NP to the guest via the nuclear Overhauser effect (NOE) effect (Figure 12B). Taking inspiration from the experiments used for target identification in drug discovery, Rastrelli and Mancin demonstrated that SAM@NPs with molecular recognition abilities could be used as selective “saturation transducers” in NOE-pumping,^158^ saturation transfer difference (STD),^158^ waterSTD^159^ and high-power waterSTD^160^ experiments, whereby the mixture spectrum is edited to retain only the signals of the guest species. Advantages of this approach include elimination of false positives and the possibility to identify even unknown analytes. In this approach, SAM@NPs not only provide a source of magnetization/saturation that is transferred to analytes that interact effectively with the monolayer, but SAM@NPs also make the transfer process efficient due to their slow tumbling rate. Accordingly, limits of detection down to 10 μM were reached by exploiting SAM@NP-silica NP composites.^161^ The method was applied to the detection of salicylate, tyramine and other neurotransmitters or psychoactive amines, biogenic amines.

In all the examples described above, the monolayer was responsible for a change in properties that could be exploited for signaling. However, other mechanisms can also be utilized, such as interparticle coupling of surface plasmons upon aggregation for SAM@NPs larger than 3 nm. In the sensing systems based on this effect, the analyte acts as a cross-linker (or as an aggregation inhibitor) promoting the formation/disruption of NP aggregates through interactions with either the NP core or specifically designed recognition sites in the ligand shell. This mechanism has been widely used in sensing systems for detecting metal ions, inorganic anions, nucleic acids, and proteins, which interact in a multivalent manner with functional groups present in SAM@NPs. For this application, we refer the reader to a more comprehensive review.^36^

### 3.3 Sensing in the Solid State Using SAM@NPs

A fundamentally different approach to SAM@NP-based sensing is their use not in solution but in the solid state, in the form of dried, thin NP films. In such films, metallic cores of the SAM@NPs are proximal but separated by insulating SAMs. However, under electrical bias, electrons can tunnel through these SAM “bridges,” endowing the nanostructured material with some electrical conductivity. Starting in 2011, Grzybowski and co-workers showed^18^ that in films made of AuNPs covered by charged ligands, the jammed SAM@NPs remain stationary under bias but the counterions around them are free to migrate, setting up dynamic ionic gradients and internal electric fields. These gradients, in turn, enable various electronic functions including current rectifiers,^18^ diodes, transistors and entire logic circuits,^162^ or even radio receivers (to listen to some Mozart music received by such a NP radio, see ref 163.). In parallel, Grzybowski and Stellacci^164^ demonstrated that when the films are composed of AuNPs decorated with ligands capable of coordinating to metal cations, the binding of such cations modifies the HOMO–LUMO gap of the Au-SAM/SAM-Au bridge, in effect, increasing the electronic conductivity of the film. They showed that this architecture allowed for the detection of various toxic cations and organometallic species with unprecedented selectivity and sensitivity—for instance, of extremely toxic methylmercury down to the attomolar level and over 18 orders of magnitude in terms of concentration. Subsequently, Grzybowski teamed up with the Yan group to integrate the electronic components with chemical sensing for cations as well as organic analytes.^165^ These so-called chemoelectronic circuits^165,166^ (Figure 13) are significantly slower to respond than semiconductor devices but, in addition to their versatile sensing abilities, offer some unique advantages in terms of material properties: they can be printed from ethanolic solutions, zapped with electrostatic discharges, or extensively deformed without losing their function (as it is extremely difficult to “break” a collection of metal spheres covered in organic “grease”).

**Figure 13:**
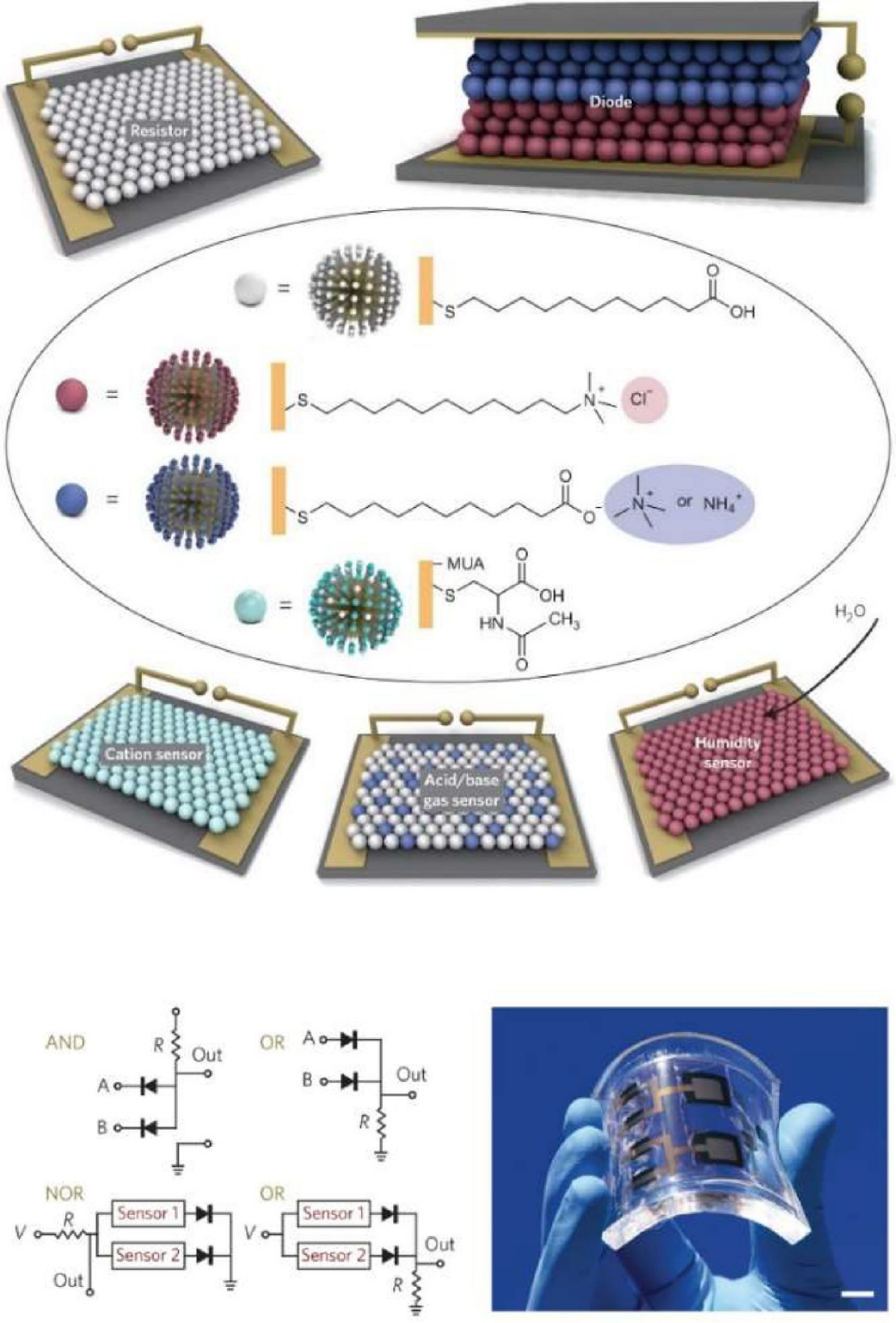
Chemoelectronic circuits based on SAM@NPs. The top portion shows the alkanethiol ligands used to coat 5.5 nm AuNPs to achieve various chemoelectronic functions. NPs functionalized with (CH_2_)_11_-N­(CH_3_)_3_^+^ ligands are surrounded by mobile Cl^–^ anions (*red*); NPs covered with HS-(CH_2_)_10_-COO^–^ ligands and surrounded by mobile N­(CH_3_)_4_^+^ or NH_4_^+^ cations (*blue*); NPs covered with HS-(CH_2_)_10_-COOH ligands (MUA) (*white*); and NPs covered with mixed SAMs of protonated MUA and HS-CH_2_CH­(NHCOCH_3_)­COOH have no mobile ions (*light cyan*). Active layers of resistors, diodes, acid/base gas sensors, cation sensors and humidity sensors were assembled using these SAM@NPs. For clarity, the illustrations show a single layer of SAM@NPs. In reality, the NP films are hundreds of nanometers thick. The scheme below shows architectures of various logic gates and gates combined with the sensing elements. The image on the lower right has an actual chemoelectronic circuit deposited and deformed on a PDMS substrate. Two golden “forks” house two sensors (each in duplicate), and the square elements are two diodes. The diodes are fully embedded in PDMS, while the sensors have their top surfaces open to the atmosphere. Scale bar is 1 cm. Figure and caption reproduced with permission from ref 165. Copyright 2016 Macmillan Publishers Limited.

## 4 Switching and Adaptation

The use of ligands that possess the ability to respond dynamically to external stimuli can result in changes to both the surface properties and the NP overall function, as anticipated in the context of catalysis regulation within SAM@NPs (see Figure 4 and related examples). These responsive ligands can undergo structural or chemical modifications in response to various external factors, such as changes in pH, temperature, light, or the presence of specific molecules.

This kind of responsiveness lies at the heart of “switchable” systems, where the ligand adaptability allows for the controlled activation or deactivation of SAM@NP functions and is deeply inspired by biological processes, where responsiveness to environmental cues is a fundamental trait. These systems were extensively reviewed previously by Klajn, Stoddart and Grzybowski;^167^ accordingly, only selected examples are illustrated here.

As the first and intuitive example—with possible relevance to bionanomedicine—we consider alteration of surface charge by pH. For example, AuNPs decorated with a mixed SAM, featuring the minority of ligands terminated in positively charged, −N­(CH_3_)_3_^+^, groups and the majority of ligands terminated in negatively charged, −COO^–^, are stable at both low and high pH but precipitate sharply at the pH where an appropriate fraction of carboxylate groups is protonated such that NP net charge is zero.^168^ By adjusting the proportion of the positively and negatively charged ligands in the mixed-charge SAM, the precipitation pH can be varied flexibly between ∼4 and ∼7 which, in turn, can regulate cell uptake. This property can be desirable to control NP uptake in heterogeneous biological environments, for example in relation to tumors, where the local pH is ∼6.5 and about one unit lower than pH of the surrounding healthy tissue. In fact, Rotello^169^ capitalized on this pH difference using zwitterionic (∓) thiols to demonstrate that AuNPs decorated with zwitterionic, pH-responsive alkoxyphenyl acylsulfonamide ligands exhibit increased uptake and cytotoxicity toward tumor cells. In their system, the SAM@NPs remain neutral at physiological pH (7.4) but become positively charged in the slightly acidic tumor microenvironment (pH < 6.5), enhancing their cellular uptake. However, we note that cationic NPs are indiscriminately cytotoxic^170–173^ and for the pH-centered approach to become a promising tool for targeted cancer therapy, it is also necessary to ensure selective action only against cancerous cells (which can be achieved with mixed-charge SAM@NPs targeting organelles known to be acidified in cancerous cells, see ref 174). For SAM@NPs operating in biological environments (including also some of the examples discussed in Section 3), an issue arises from the fact that molecular species present in the biological milieu (such as proteins, phosphates, and polyions) can interact with the monolayer, thereby altering its physicochemical nature, affecting its conformation or competing with the target molecule for binding.^17^ Therefore, strategies must be adopted to mitigate or suppress such undesired interactions when designing such SAM@NPs for a specific function. The most common approaches include the use of neutral or zwitterionic groups in the ligand structure,^175^ specific ligand organization in mixed monolayers,^176^ the introduction of hydrophilic “stealth” layers resistant to the adsorption of charged biomolecules,^17^ and size reduction to ultrasmall SAM@NPs.^177,178^ Yet, given the wide range of synthetically accessible ligand designs and emergent nanoscale effects,^8,179^ it remains difficult to anticipate whether these strategies are always effective. Future progress will require the close integration of simulations, experiments, and data-centered approaches to enable the *a priori* prediction of SAM@NP biointeractions.^180,181^

Besides pH, light serves as another potential trigger or external stimulus. While the Au core is rather inert to pH changes, light stimuli are rarely completely orthogonal and can affect both the core and the ligand shell. Indeed, optical features arising from local surface plasmon resonance and scattering overlap with chromophore absorption in most cases. It is thus not surprising that in most photoswitchable systems, particles tend to have a small size (<6 nm), to minimize these two optical phenomena. Moreover, as discussed above, Au cores can quench the excited state of molecular entities, suppressing their photochemical behavior. In addition to these elements, steric crowding also plays a role in photochemical processes involving SAM@NPs. This element was investigated in a number of systems bearing pendant AB units; AB-derivatives are established tools to create photoresponsive surfaces and materials leveraging the ability of AB to undergo reversible photoisomerization between its *trans* and *cis* forms. While several studies have explored NP systems functionalized with AB-terminated alkanethiolate molecules, these systems often exhibit minimal photoreactivity under ultraviolet (UV)/visible light due to the formation of densely packed SAMs and quenching by the NP core. In order to tackle these problems, Knoll capped AuNPs with an unsymmetrical AB disulfide, bearing a dodecyl spacer between the AB and the disulfide.^182^ Upon exposure to UV light, these AB molecules isomerize from *trans* to *cis*. The photoisomerization process is highly efficient, similar to free AB molecules in solution, indicating no evident effects of steric hindrance coming from nearby ligands. The efficient isomerization is also attributed to the disulfide employed in preparing the SAM@NP, which is not symmetric and bears only one AB moiety. This asymmetry leads to the formation of free volume within the monolayer, as only 50% of the components bear a terminal AB. Moreover, the loosely packed molecular tails resulting from the curved colloidal gold surface further facilitate isomerization, as discussed in greater detail by Klajn, Grzybowski et al.^167^ In the Knoll study, isomerization is accompanied by sedimentation of the NPs in toluene, attributed to differences in solvation between the AB isomers. Understanding how photoswitches (and in particular AB) can be operated efficiently in SAM@NPs has inspired the development of mechanisms for remote spatiotemporal control over NP aggregation and assembly (see Section 5).

Additional insight on the photoreactivity of AB on Au nanomaterials was reported more recently by Ma,^183^ who explored the dynamic photoresponsive behavior of AB-functionalized Au nanomaterials (Au@AB). The authors examined six different Au@AB combinations, utilizing two AB derivatives (rigid biphenyl-linked and flexible alkoxyl chain-linked) and three types of Au substrates: planar Au(111), and curved Au_102_(SR)_44_ and Au_25_(SR)_18_ clusters. Using a reactive MD model, the authors investigated the *cis-*to*-trans* isomerization process, which was driven primarily by the torsion of the C–N = N–C dihedral angle, with minor contributions from an inversion pathway. Findings indicated that substrate curvature and AB backbone flexibility significantly influence switching behavior. Flexible ABs switch faster on curved NPs, while rigid ABs switch more rapidly on planar substrates due to π–π stacking interactions. The study demonstrates that both AuNP size and AB type jointly determine the collective switching behavior, and emphasizes the importance of carefully designing photoswitchable SAM@NPs considering the multilength scale systems nature of structure–function relationships.

A feature of most AB-functionalized AuNPs is that they are soluble in organic solvents, rather than water, owing to the apolar character of *trans-*AB. Klajn addressed this challenge by coadsorbing hydrophobic AB ligands with water-soluble ligands, such as those terminated with oligo­(ethylene glycol) or charged groups.^184^ A distinctive functionalization method was developed to control the molar ratio of these ligands on the AuNPs, enabling precise tuning of their surface composition. MD simulations revealed two distinct supramolecular architectures, enhancing the aqueous solubility of the AuNPs. These AB-coated AuNPs retained their light-switchable properties, exhibiting efficient reversible photoisomerization in water, which expands their applicability in biological environments. Importantly, the choice of background ligands significantly affected the kinetics of AB switching, with hydroxy-terminated ligands accelerating back-isomerization by over 6000-fold, due to interligand hydrogen bonds reducing the double bond character of the diazo unit within the monolayer-confined environment (Figure 14A).

**Figure 14:**
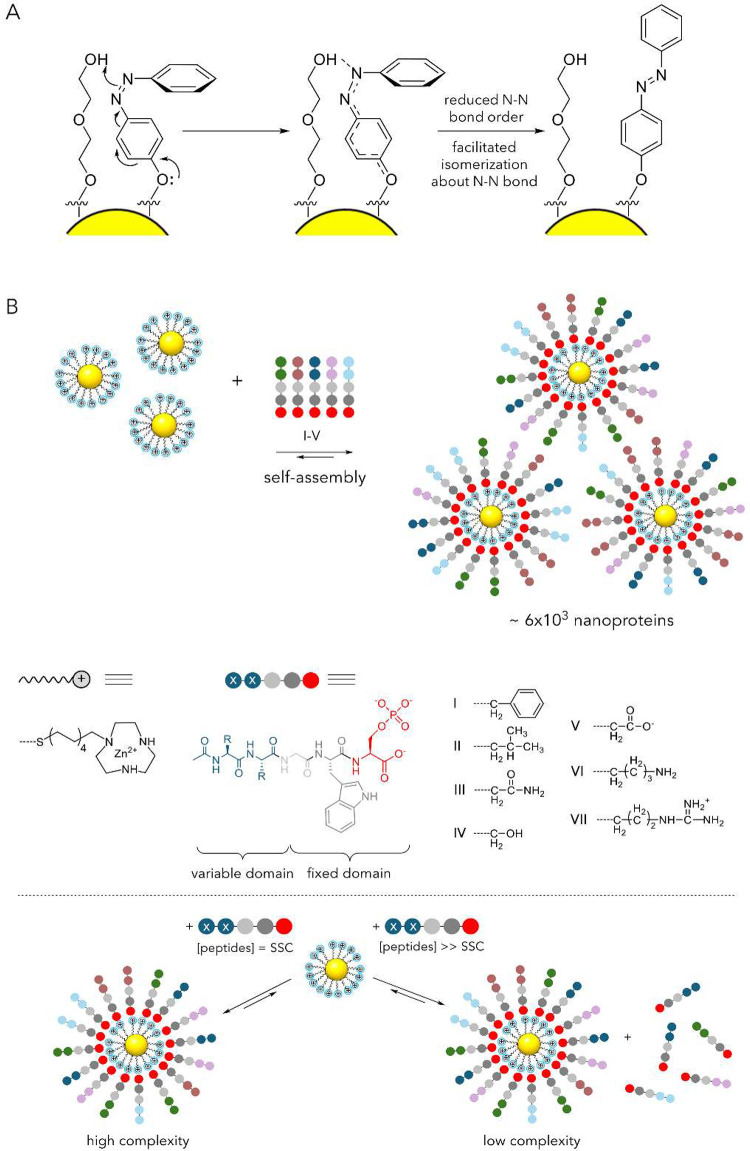
(A) Proposed mechanism for back-isomerization of *cis*-AB photoswitching on SAM@NP, assisted by a neighboring hydroxy group present on the background ligands. Reprinted with permission from ref 184. Copyright 2018 American Chemical Society. (B) Example of dynamic nanoproteins formed through the self-assembly of small peptides on the surface of SAM@NPs. Peptide surface complexity arises as a function of the peptide concentration (SSC, surface saturation concentration). Reprinted with permission from ref 185. Copyright 2016 Royal Society of Chemistry.

AB-containing thiols are not the only photoswitches that can impart light-responsiveness to SAM@NPs. For example, Sashuk’s group showed that AuNPs coated with the recently discovered donor–acceptor Stenhouse adduct (DASA) chromophores, previously considered nonswitchable due to emerging strong electrostatic stabilization of its closed form, can be made switchable by dilution with hydrophobic coligands. The polarity of such SAM@NPs and aggregation state with increasing DASA content changes nonlinearly during light irradiation due to the decrease in the degree of isomerization, the decrease in the content of the hydrophobic background coligand, and the increase in the content of the DASA ligands retaining the native form.^186^

Besides photoswitches, light has also been used in combination with photocaged moieties to trigger SAM@NP modifications. In a seminal example, Prins and Scrimin investigated the rapid and efficient exchange reaction of thiol-containing peptides in a cationic SAM on AuNPs at low micromolar concentrations in water.^187^ The strong binding affinity of anionic peptides to the cationic surface significantly increases the local concentration of peptides incorporating both anionic and thiol groups, facilitating the exchange reaction—which otherwise tends to be kinetically slow and requires high thiol concentrations. By using a photolabile protecting group on the thiol moiety, the exchange reaction can be induced photochemically. These findings showcase how light can be used for the precise and controlled synthesis of heterofunctionalized SAM@NPs, with potential for applications in nanomedicine and diagnostics due to the high biocompatibility and functional versatility of water-soluble AuNPs.

The use of redox stimuli to control SAM@NPs has also been reported. An attractive feature is the simplicity by which they electronically responsive nanosystems can be interfaced with modern technology. NPs have been functionalized with a variety of electroactive ligands, ranging from simple redox-active units to complex bistable interlocked structures and related architectures.^167,188,189^ In the context of supramolecular interactions and nanostructured devices, Huskens achieved reversible redox-controlled attachment of NPs to a macroscopic 2D surface using a molecular printboard.^190^ Specifically, ferrocenyl-functionalized poly­(propyleneimine) dendrimers act as multivalent recognition units for the adsorption and desorption of (β-CD)-functionalized AuNPs onto a β-CD SAM. The process leverages the electrochemical oxidation of ferrocenyl groups to induce the desorption of NPs from the 2D-SAM. The attachment and detachment of β-CD-functionalized AuNPs (β-CD-AuNPs) and larger silica NPs (60 nm diameter, β-CD-SiO_2_) were monitored using surface plasmon resonance (SPR) spectroscopy and electrochemistry. The authors demonstrated that electroactive dendrimers enable precise control over nanostructure assembly and disassembly on molecular printboards, potentially applicable to various nanostructures and surface modifications. The employed [ferrocene•CD] recognition motif is highly reliable. For example, Frasconi and Mazzei investigated the reversible assembly of β-CD-AuNPs on mixed SAMs composed of redox-active ferrocenylalkanelthiols and *n*-alkanethiols on 2D Au surfaces.^188^ The surface coverage and spatial distribution of β-CD-AuNPs are controlled by the SAM composition. SPR spectroscopy was used to monitor the binding and release of β-CD-AuNPs from the modified surface, regulated by the redox state of the ferrocene in the SAMs. The authors demonstrated the electrochemically induced uptake and release of β-CD-AuNPs, facilitated by supramolecular interactions with the ferrocene-functionalized surface. The inclusion of a suitable AB guest able to interact competitively with β-CD imparts a second means to control the system in this case using light. This dual stimuli-responsive system serves as a proof-of-principle for an active molecular plasmonic device capable of performing elementary logic gate operations based on optical and electrical inputs. Additionally, the system potential for sensing and drug delivery applications is explored by controlling guest molecule association within the β-CD cavity, thereby highlighting its versatility and potential in nanotechnology and biomedicine. Redox-switched SAM@NP systems have been further developed for controlling NP assembly in solution, as discussed further in Section 5.

In the context of SAM@NPs, adaptation can exploit the same noncovalent interactions that drive self-assembly to promote spontaneous exchange processes on the 3D monolayer surface. These surfaces can adapt to environmental changes, mimicking the complexity and functionality of natural proteins. For example, the high affinity of SAM@NP **1**•Zn^2+^ system (the same discussed before in Section 3) for small oligoanions was explored^185^ to create dynamic peptide surfaces by self-assembling with the monolayer small peptides comprising a phosphorylated Ser-residue for binding to SAM@NP **1**•Zn^2+^ (Figure 14B). This method enables easy tuning of the peptide surface complexity by varying the chemical nature and ratio of the added peptides. The dynamic nature of the surface permits adaptation of the surface to changes in the environment and offers the possibility of developing self-selection processes.

## 5 Programmable NP Assembly

When incorporated into superstructures and networks, the distinctive properties of NPs are modulated and amplified, collective properties not observed for isolated NPs emerge, and multicomponent systems that combine the properties of more than one NP type become feasible.^191–193^ Furthermore, hierarchical NP superstructures spanning multiple length-scales and composites that integrate NPs with bulk materials or interfaces will be crucial for putting nanoscale properties to use in technologies, from microelectronics to living systems.^194–197^

Solution-processable SAM@NPs allow us to consider the exciting prospect of bottom-up construction strategies that apply rational principles of molecular synthetic chemistry to chemically active nanometer-sized building blocks. Their multicomponent makeup, and the size-similarity of NP core, surface-bound ligands, and unbound matrix constituents engenders a complex landscape of coupled forces that influence NP interactions and, hence, assembly.^198,199^ If this landscape can be reliably navigated, the convergence of interaction forces and thermal energy at this size scale is arguably optimal for constructing persistent superstructures that also exhibit dynamic properties—it is no coincidence that the building blocks for dynamic functional structures in living systems have nanometer dimensions.

When the influence of the ligand shell is insignificant, SAM@NPs behave as “hard particles”. Assembly is directed by isotropic dispersion interactions, entropic solvent depletion forces and entropic ordering of the constituents. Under precisely tuned conditions, kinetically trapped amorphous aggregates can be avoided, leading to exquisite crystalline NP superlattices.^192,200^ In practice, the ligand shell structure and dynamics often have an inescapable influence on the assembly outcome.^201–207^ However, the superstructure parameters remain inextricably linked to the size and shape of the constituent NPs. External magnetic or electric fields offer some degree of independent control over assembly state and structure,^208^ but only for core materials that respond to these stimuli.

Engineering interparticle interactions through rational design of the ligand shell molecular composition, supramolecular structure and dynamics is emerging as a powerful strategy for programming SAM@NP assembly, decoupled from the characteristics of the underlying core. In doing so, the collective and emergent properties of the resultant superstructures are encoded at the molecular level, through both the structural features of the interparticle linkages and by the molecular-level events that lead to assembly. Structural features spanning several length scales (Figure 15A–C) can therefore be dictated by this chemically informed approach to instruct architectural and dynamic characteristics of assembly superstructures. Specifically:

A.1) **Linkage atomic detail**. The molecular structure and chemical bonds responsible for connecting between each NP building block.
A.2) **Linkage architecture**. The relative arrangement of reactive or interactive groups that defines how the molecular components engage two or more NP building blocks.

B.1) **Nanoscale local structure**. The identity, valency and spacing of neighbors to each NP building block.
B.2) **Nanocomponent composition**. The identity of the constituent NP building block(s) and their relative proportions across the material (or a domain thereof).

C.1) **Mesoscale structure**. Dimensionality, porosity, grain size and degree of order on nanometer to micrometer length scales.
C.2) **Macroscopic structure**. Material form (e.g., monolithic solid, powder, thin film) and patterning on millimeter or longer length scales.

**Figure 15:**
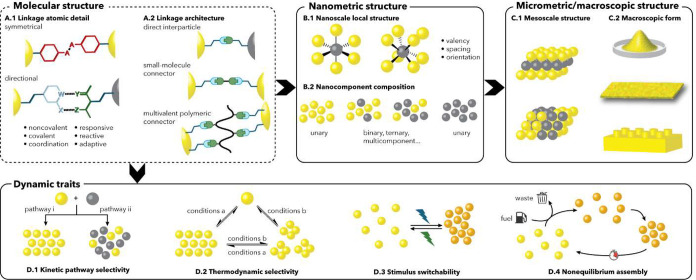
Programmable structural (B, C) and dynamic (D) characteristics of SAM@NP assemblies that can be encoded in the molecular length-scale features of the surface-bound ligands on AuNPs (A).

Even with precise control over each of these structural factors, static assemblies have fixed properties. Excitingly, dynamic traits of the molecular-level interactions and reactions between NP-bound ligands are also conferred on NP superstructures, leading to programmability in what is produced, where and when. The pioneering examples we examine here exhibit dynamic behaviors in one or more of the following ways (Figure 15D):

D.1) **Pathway-dependent assembly** through kinetic selectivity.
D.2) **Thermodynamically governed assembly** outcomes that are pathway-independent.
  In favorable cases, it becomes possible to apply external stimuli to manipulate kinetic and/or thermodynamic factors to achieve:
D.3) **Stimuli-responsive switching** of assembly state.
D.4) **Nonequilibrium assembly states** maintained by dissipative processes.

As with each of the functions discussed in Sections 2–4, the behavior of SAM@NP reaction and interaction sites is strongly influenced by the distinctive monolayer environment.

By definition, SAM@NP assembly also entails bringing the NP cores into close proximity, potentially introducing energetically and electronically significant interactions between the inorganic components. For example, strong attractive dispersion forces between heavy element cores such as gold pose particular challenges for creating switchable and adaptive assemblies.

Modulating the charge state of acidic or basic functional groups in the ligand shell (see also Section 4) allows switching between NP dispersions and assemblies. Electrostatically stabilized dispersions in polar solvents can be reversibly converted into amorphous aggregates or 2D films by adjusting solution pH.^209–215^ Notably, hysteretic behavior is often observed, which is a consequence of the strong repulsive electrostatic interactions and attractive dispersive forces that are at play.

The surface charge of SAM@NPs is subtly sensitive to multiple structural and environmental factors (see Section 2 for a theoretical description of some of these factors), several of which can be manipulated to direct assembly. For example, diluting permanently charged cationic ligands with hydrophobic ligands creates amphiphilic SAM@NPs that are very sensitive to the composition of the continuous phase.^216^ Only on adding a certain proportion of methanol to a biphasic dichloromethane/water system, these NPs undergo self-assembly, migrating from the bulk organic phase to form an equilibrium 2D film with a densely packed hexagonal structure. Zeta potential measurements suggest that the crucial role of methanol is to modulate SAM@NP surface charge by influencing counterion dissociation energy.

Amphiphilic SAM@NPs can also be created from neutral ligands and have been demonstrated to undergo thermoresponsive assembly/disassembly.^217^ Dehydration of hexa­(ethylene glycol)-derived surface ligands on increasing temperature generates solvophobic forces that drive SAM@NP self-assembly. Although the individual ligand molecules show similar thermoresponsive aggregation phenomena, the SAM@NP behavior is unique to the nanostructure—with significantly higher characteristic transition temperatures that are dependent on both NP size and molecular structure of the ligand end group.

The strength, long-range influence and universality of electrostatic dispersion and solvophobic forces limit their utility for precisely encoding multicomponent superstructures. Molecular recognition motifs that exploit short-range noncovalent interactions offer structurally encoded selectivity, directionality, temperature-sensitive kinetics and, potentially, responsiveness to other external stimuli,^218–225^ although arguably against a trade-off in ligand structural complexity and synthetic burden. Pioneering studies established that NPs with ligands incorporating a molecular recognition motif could be assembled using each possible linkage architecture (Figure 15A.2): direct association between ligands bearing complementary recognition partners;^226^ bridging via a complementary ditopic small-molecule connector;^227^ or complementary multivalent macromolecular cross-linker.^224^ These early studies demonstrated the benefits of pairwise specific recognition motifs for selective linking of distinct building blocks,^227^ and conformational flexibility for masking the inherent polydispersity of NP samples and for assembling adaptive superstructures.^224^

Stimuli-responsive molecular recognition motifs enable remote control over NP assembly state (Figure 15D.3) by modulating high-fidelity selective interparticle linkages. Redox-switched aggregation–dispersion of AuNPs was achieved by including a small proportion of redox-active electron-rich guests (tetrathiafulvalene, TTF) in the ligand shell, combined with a multivalent polymeric cross-linker bearing complementary electron-poor macrocyclic hosts (cyclobis­(paraquat-*p*-phenylene), CBPQT^4+^-side-chain functionalized poly­(methyl methacrylate)).^228^ At stoichiometries close to 1:1 host:guest, linear chain-like structures formed at short time periods, evolving into extended cross-linked precipitates at longer times. NP redispersion was triggered by chemical or electrochemical oxidation of the TTF guest to its dicationic form, thus disrupting the attractive [ligand-host•polymer-guest] molecular recognition. The aggregated state was restored on reduction back to the neutral TTF state. Although the switchable molecular recognition behavior of the [TTF•CBPQT^4+^] complex is well understood,^229^ the SAM@NP environment strongly modulates the redox switching potential^189^ and defines the multivalent linkage architecture (Figure 15A.2). The programmable assembly behavior is consequently dictated by a combination of thermodynamic and kinetic factors that are unique to the makeup of the nanoscale and macromolecular assembling components.

The well-developed understanding of CBPQT^4+^ host–guest chemistry^229^ could be translated to this context to achieve remarkably selective capture and release of distinct NPs actuated by external stimuli (Figure 16A).^228^ Redox-inactive dioxynaphthalenes (DNP) have affinity for CBPQT^4+^ that is intermediate between the charged and neutral TTF states (*i.e*. association constants [TTF•CBPQT^4+^] > [DNP•CBPQT^4+^] > [TTF^2+^•CBPQT^4+^]). Consequently, from an equimolar mixture of AgNP-TTF_**(0.05)**_ and AuNP-DNP_(0.05)_, the CBPQT^4+^-side chain functionalized polymer selectively aggregates the silver NPs (AgNPs), leaving a characteristically red-colored solution of AuNP (Figure 16A, right). Oxidation of the TTF units resulted in almost complete redispersion of the AgNPs as AgNP-**TTF**_(0.05)_^2+^, coincident with cross-linking and precipitation of the AuNPs instead, leaving a pale yellow solution characteristic of colloidal silver (Figure 16A, left).^228^

**Figure 16:**
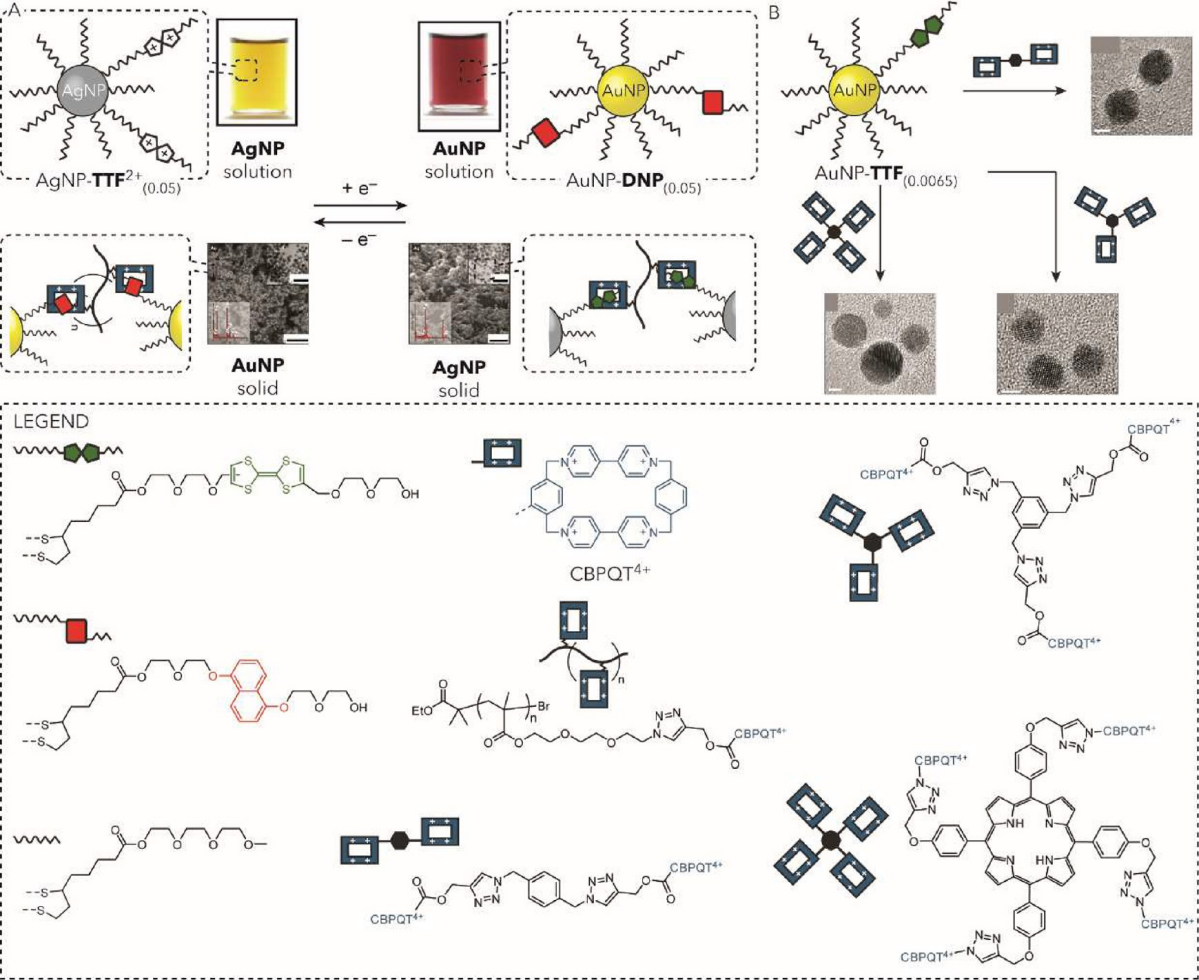
Redox-controlled programmable NP assembly. (A) Nanocomponent compositional selectivity and (B) cluster valency are encoded in molecular recognition interactions between electroactive ligands and multivalent molecular connectors. Numbers in brackets indicate the mole fraction (χ) of functional ligands in each NP-bound monolayer; *n* ∼ 160. Scale bars: (A) 300 nm (left), 30 nm (inset, left), 200 nm (right), 30 nm (inset, right); (B) 2 nm (all). Adapted with permission from ref 228, Copyright 2009 Macmillan Publishers Limited (A) and ref 230, Copyright 2009 American Chemical Society (B).

Assembling structurally defined discrete NP superstructures is arguably a more demanding challenge than producing extended amorphous (or even crystalline), aggregates because the former requires controlling the number and relative disposition of interparticle linkages across a nanoscale surface using only molecular length scale structures.

Using the [TTF•CBPQT^4+^] recognition motif, redox-switchable NP dimers, trimers and tetramers were assembled from AuNPs bearing TTF ligands on combination with di-, tri- or tetravalent CBPQT^4+^ linkers, respectively (Figure 16B).^230^ To minimize the likelihood of cross-linking between discrete multimers, it was critical to maximize the population of NPs bearing only one guest ligand (AuNP-TTF_(0.0065)_). This was only possible by statistical means, preparing samples in which the majority of NP ligand shells contained no guest ligand at all. Assembly yields were consequently very low and the discrete target structures were accompanied by poorly defined aggregates.

Nevertheless, high resolution TEM imaging revealed multimer configurations consistent with the computed dimensions of each multivalent molecular connector, demonstrating the capability of even small-molecule structures to define valency within superstructures formed from nanoscale building blocks.

A degree of valency control can alternatively be achieved through appropriate design of anisotropic NP cores to achieve regioselective surface attachment of difunctional molecular cross-linkers. For example, Au/Fe_3_O_4_ particles presenting chemically distinct “steric hindrance” (Fe_3_O_4_) and cross-linkable (Au) surface domains can be driven to preferentially form NP dimers, trimers or tetramers on reaction with dithiol linkers.^231^

High-fidelity molecular recognition has also proven a powerful means of programming the assembly of polymer brush-coated NPs in the form of either host–guest recognition at the periphery of end-tethered abiotic polymers or hybridization between end-tethered synthetic oligonucleotides.^232^ Polymer brush coatings bring attributes of macromolecular linking structures (*vide supra*) into the monolayer and display distinctive behaviors compared to small-molecule SAM@NPs.^232^ However, it is valuable to note that, in these contexts too, surface-specific cooperative effects such as dynamic ligand bundling have an inescapable influence on intermonolayer bonding.^233^ Here too, therefore, a systems-level approach is required to rationalize the influence of structural and dynamic factors spanning several length scales on the particle assembly process and superstructure characteristics.^234^

While noncovalent molecular recognition motifs are labile under virtually all circumstances and have a tendency to be structurally promiscuous, covalent bonds are strong and structurally precise. Taking advantage of the mature synthetic methodology for making covalent bonds through chemospecific reactions, covalent linking of solution-processable ligand-coated NPs would appear to be attractive for bottom-up superstructure assembly. However, the reactions commonly applied for NP conjugation tend to be kinetically controlled^3,6,7^ and, when used to connect NPs, can amplify the effects of NP polydispersity and typically offer few options for tuning structural parameters or superstructure behavior. Exceptions include situations in which a ligand bearing a reactive functional group can be installed in selective positions within the monolayer through spatially localized place-exchange at polar defects,^236^ or using surface-masking approaches.^237^

Dynamic covalent reactions combine the favorable features of equilibrium processes with the stability and structural precision of covalent structures and the programmability of covalent synthetic strategies.^238,239^ Dynamic covalent NPs (DCNPs), in which the surface-bound ligands incorporate a functional group capable of reversible covalent modification,^240,241^ are consequently emerging as instructable nanoscale building blocks that can be selectively modified according to the rational principles of molecular synthetic chemistry.

Assembly and disassembly of covalently linked NP networks was first demonstrated using rapidly equilibrating boronate ester links formed between NP-bound boronic acids and molecular catechols.^242^ By contrast, dynamic covalent exchange of hydrazones typically requires the action of an appropriate catalyst to occur at appreciable rates, offering on-demand control. A pair of DCNPs was constructed by coating noble metal cores (Au and Pd) with hydrazone-terminated ligands in which the reactive site was connected via either the nucleophilic hydrazido end or electrophilic benzylidene end (Figure 17A, orange and blue respectively).^105,243^ These two chemically complementary DCNPs do not interact in solution until an acid catalyst is introduced, triggering dynamic covalent hydrazone exchange. Subsequently, aggregates emerge, growing to incorporate all NPs in extended networks.^243^ Interparticle connectivity is defined by the directional hydrazone links that are formed in a chemospecific covalent reaction. Thus, the two NP constituents are intimately mixed throughout the network, with no possibility of phase-segregated domains.^105^ The networks are also endowed with the dynamic characteristics of the atomic-level links. Aggregate growth responds (Figure 15D.2) to the presence of a small-molecule monotopic aldehyde to produce discrete colloidally stable superstructures with equilibrium sizes that decrease on increasing concentration of the capping agent.^243^

**Figure 17:**
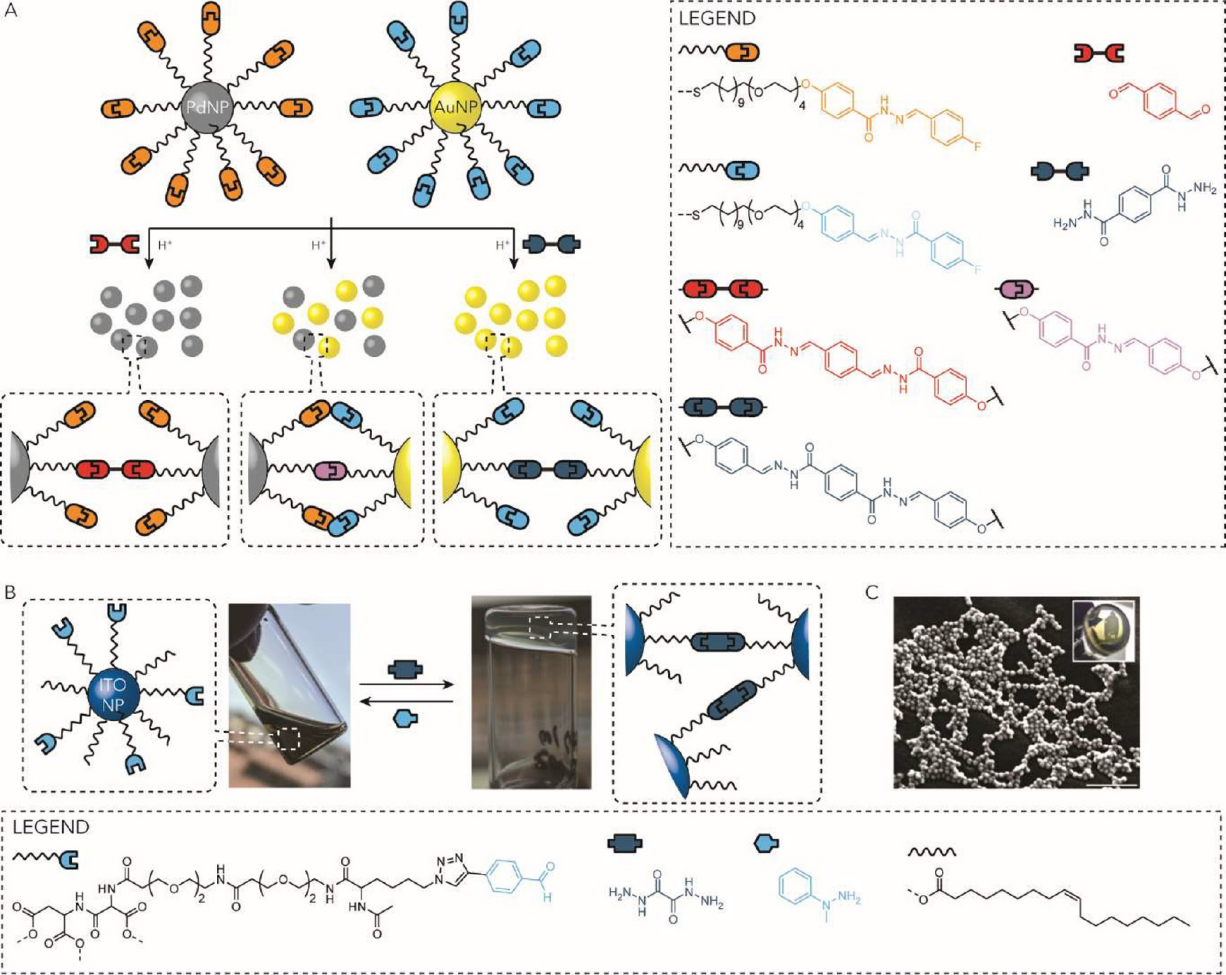
Dynamic covalent linking for programmable SAM@NP assembly. (A) Compositionally selective assembly from a mixture of dynamic covalent NP building blocks, instructed by chemospecific dynamic covalent linking. Under each selective assembly pathway, aggregate composition is tunable by adjusting kinetic selectivity between multiple molecule–SAM@NP and SAM@NP–SAM@NP reactions operating under dynamic covalent exchange conditions. (B) Reversible formation and dissolution of NP gels by formation and disruption of dynamic covalent links. (C) SEM image confirming persistence of discrete NP constituents within the gel network. Scale bar: 100 nm. Adapted with permission from ref 244. Copyright 2020 American Chemical Society (B, C).

The one mixture of cross-reactive DCNPs could be directed down different assembly pathways in response to molecular instructions (Figure 15D.1) in the form of complementary ditopic small-molecule connectors. Gold-enriched aggregates were selectively produced on introducing a ditopic nucleophilic connector; palladium-selective assembly was achieved using a ditopic electrophile (Figure 17A).

Kinetic models of the covalent exchange reaction networks, parametrized by experimentally measured rate constants, were capable of rationalizing—and guiding the optimization of—assembly compositional selectivity. It was thereby revealed that selectivity is governed by the influence of the SAM@NP environment on reaction kinetics, which in turn depends on mechanistic and structural factors on both molecular and nanometer length scales.^105^

Using dynamic covalent SAM@NPs as building blocks, new types of bulk materials can be constructed, that combine properties of both the nanometric component and the programmable molecular linkages. SAM@NPs comprising tin-doped indium oxide (ITO) cores coated with a mixed-ligand monolayer including aldehyde-terminated ligands formed phase-separated gels over several days after introducing a complementary bis-hydrazide small-molecule connector (Figure 17B).^244^ In contrast to other gelation strategies, the chemospecific molecular length-scale links produce a thermodynamic NP gel,^245^ in which the individual SAM@NP building blocks are preserved as discrete structures (Figure 17C). Furthermore, the programmable interparticle bonds enabled chemically responsive cycling between dispersed and gel phases (Figure 15D.3), giving rise to switchable optical properties on account of coupling between the plasmonic NP cores in the gel state.^244^ In addition to dynamic reconfigurability, thermodynamic gels potentially offer improved predictability and rational programmability of phase behavior and structure encoded by molecular interactions with the SAM@NP ligand shell, compared to kinetically controlled NP gelation strategies.^245,246^

Building on early examples of transient NP self-assemblies maintained by an energy flux,^247^ programming NP assembly state using ligand shells that respond to dissipative (photo)­chemical processes opens up prospects for harnessing nanoscale properties in animate materials and networked systems. Photochromic switches are well-established devices for modulating molecular properties,^248^ have been used to control access for small molecules into the SAM@NP microenvironment (Section 2) or to control SAM@NP properties (Section 4), and can equally be applied to modulate interparticle interactions.^249^ Photoswitch structure determines the wavelengths required to trigger isomerization and the time scale for thermally activated relaxation from the metastable to ground state isomers. It is thereby possible to design a variety of programmable nanosystems ranging from transient states that are maintained only during irradiation, to metastable aggregates that persist for a tunable time-period.

In a seminal example, metal NPs coated with AB-containing ligands aggregate under UV irradiation as a result of solvophobic forces and attractive dipole–dipole interactions between the polar *cis*-AB isomers.^98,182^ Solvent-stable dispersions are rapidly recovered in the dark as a result of thermal relaxation to regenerate the more stable *trans*-AB ligand isomer (Figure 18A). Metastable micron-sized crystals could be assembled using a dithiol photoswitch structure capable of forming all-covalent interparticle cross-links. However, crystallinity and reversibility were only achieved concurrently for a narrow range of photoswitch surface density and solvent polarity,^98^ again illustrating the multiparameter sensitivity of interparticle forces and their influence on multi length-scale structures.

**Figure 18:**
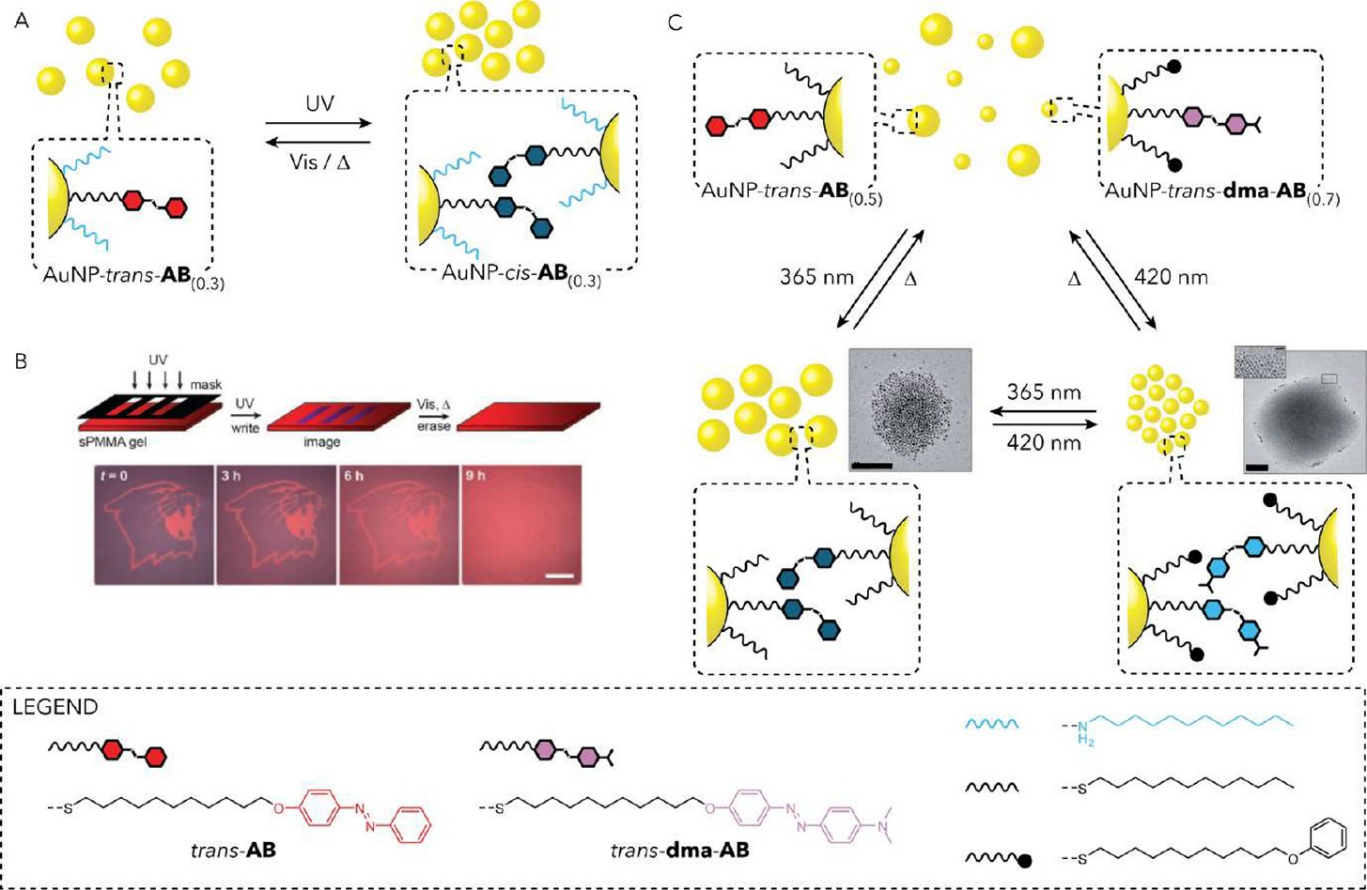
Light-controlled programmable NP assembly. (A) Photoisomerization of AB-functionalized ligands induces NP aggregation in apolar solvents on account of the large dipole moment for the metastable *cis*-AB isomer. Redispersion occurs on recovery of the thermodynamically preferred *trans*-AB isomer, triggered by irradiation with visible light or by thermal relaxation. (B) Transient surface patterning generated by plasmonic coupling in metastable NP aggregates generated within a poly­(methyl methacrylate) gel on irradiation through a photomask. Images show the gradual erasure of an image over time. Image lifetime is programmable by the mole fraction (χ) of photoswitch in the monolayer (here χ = 0.3). Adapted with permission from ref 250. Copyright 2009 WILEY-VCH GmbH. (C) Compositionally selective NP aggregation programmed by wavelength-selective photoisomerization of AB photoswitch ligands. In a mixture of 2.5 nm AuNPs functionalized with *N*,*N*-dimethylamine-substituted azobenzene (dma-AB) ligands and 5.5 nm AuNPs functionalized with AB ligands, irradiation with blue light selectively generates the *cis*-dma-AB configurational isomer, resulting in metastable aggregates of 2.5 nm AuNPs. Irradiation with UV light restores the *trans*-dma-AB isomer and generates *cis*-AB, resulting in metastable aggregates selectively composed of the 5.5 nm AuNPs. Numbers in brackets indicate the mole fraction (χ) of functional ligands in each NP-bound monolayer. Adapted with permission from ref 251. Copyright 2015 WILEY-VCH GmbH. Scale bars: (B) 1 cm; (C) 100 nm.

Embedding photoswitchable SAM@NPs in a polymer gel spatially fixed the transient aggregates and extended their lifetimes to several hours. This allowed the visible color changes generated by plasmon coupling between the metal NP cores in the aggregated state to be harnessed as a photoresponsive self-erasable and reusable ink (Figure 18B). Multicolor images could be created by modulating the irradiation time, while the erasure time was sensitive to the surface density of photoswitches, exposure to visible light or temperature.^250^

Dipole–dipole interactions are nonspecific and nondirectional electrostatic forces, limiting the options for engineering selectivity through the interactions themselves. An innovative solution used wavelength-selective isomerization of structurally distinct AB photoswitches to achieve constitutionally selective assembly from a binary mixture of different sized AuNP (Figure 18C).^251^

While light offers a waste-free energy source that can be applied with spatiotemporal precision and wavelength selectivity, chemically powered systems can be entirely autonomous and can exploit virtually limitless selective and orthogonal interactions encoded in molecular structure and reactivity^252^ to assemble superstructures that are defined by the kinetic features of the coupled reaction network.^253^

Remote-controlled actuation or autonomous NP assembly–disassembly has been achieved by coupling charge-switchable NP ligand shells (*vide supra*) with photoacids or out-of-equilibrium pH oscillating reactions.^254–257^ In these examples of assembly under dissipative conditions,^258^ the ligand charge state responds to the transient environmental changes so that the [NP–solvent] system is locally equilibrated. A fundamentally different approach to chemically powered NP assembly is to harness nonequilibrium ligand structures that transiently direct (or disrupt) the formation of dissipative superstructures (Figure 15D.4).^259^

Several dissipative supramolecular assemblies have been generated in aqueous environments based on the interconversion between carboxylates and hydrolytically unstable esters or anhydrides, powered by carbodiimide reagents.^260^ This reaction cycle (Figure 19A) was applied to silicon nanocrystals coated with carboxylate-terminated ligands.^261^ On addition of the carbodiimide fuel, optically transparent NP dispersions became turbid—indicating aggregation caused by conversion of the charged ligands to neutral, hydrophobic *N*-hydroxysuccinimido (NHS) esters. After a period of time that was tunable between 4–48 h according to the fuel concentration, charge-stabilized NP dispersions were autonomously recovered as a consequence of the ligands returning to their carboxylate form.^261^

**Figure 19:**
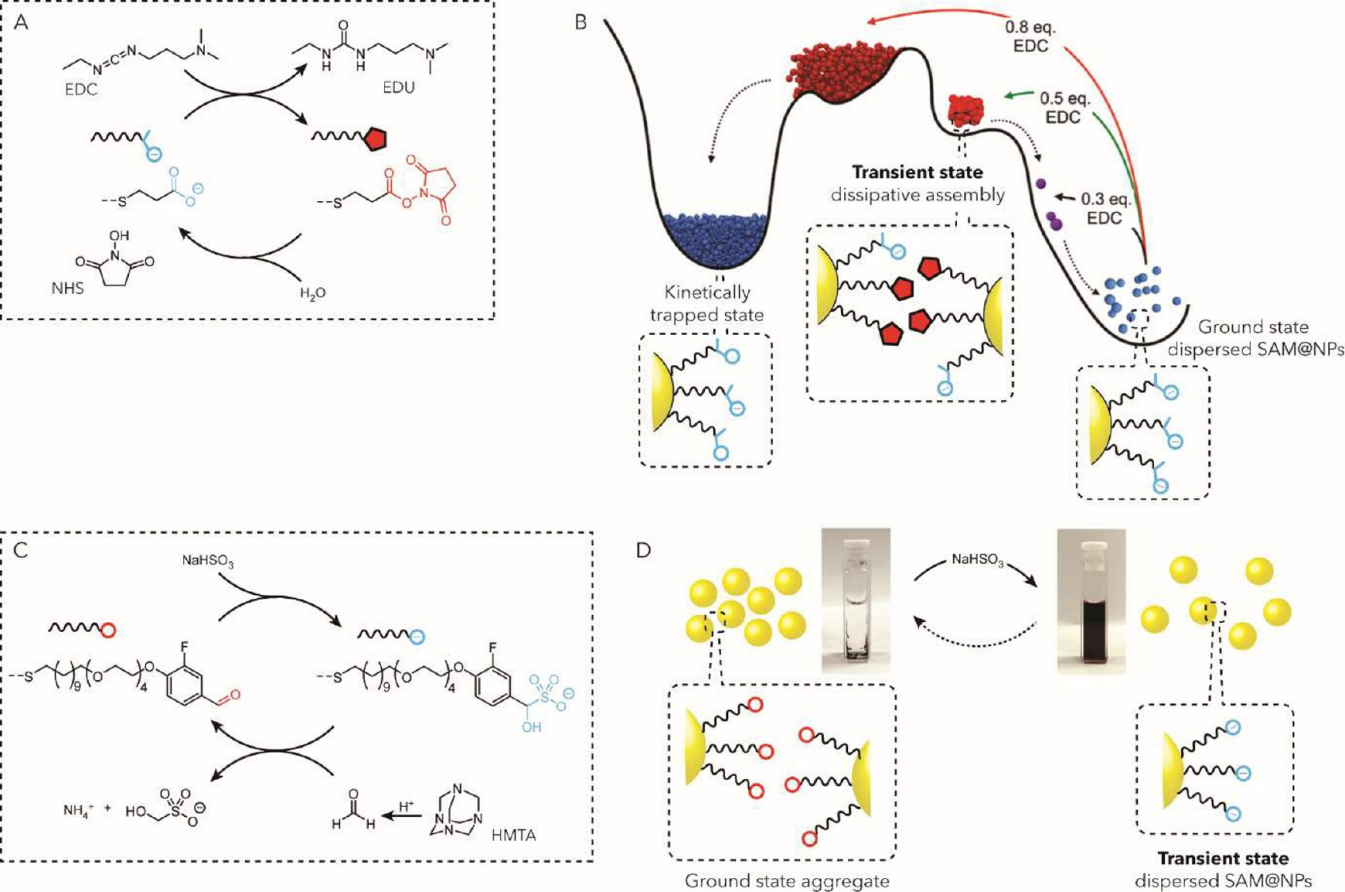
Programmable transient SAM@NP assembly/disassembly under nonequilibrium conditions. (A, B) Transient formation of dissipative SAM@NP assemblies in water on generating neutral NHS ester ligands fueled by carbodiimide (EDC) conversion to urea (EDU) waste. The kinetically trapped state is observed for Au cores at high fuel concentrations and can be dispersed by increasing pH, suggesting that both van der Waals attraction between metal cores and ligand charge state play a role. The cartoon representation of surface charge state in the dispersed and kinetically trapped states is purely schematic. (C, D) Transient SAM@NP dispersion in water on generating negatively charged α-hydroxysulfonic acid ligands, which are returned to neutral aldehydes under dissipative action of HMTA decomposition to produce formaldehyde. Adapted with permission from ref 262, Copyright 2019 American Chemical Society (B) and ref 263 under a Creative Commons CC BY license, Copyright 2023 Wiley-VCH GmbH (D).

In an interesting application, imaging of the fluorescent silicon nanocrystals by confocal microscopy revealed that the metastable state comprised micron-sized clusters that were resistant to cell uptake, which proceeded rapidly once the dispersed state was regenerated.^261^ When the same reaction cycle was applied to gold-cored carboxylate terminated SAM@NPs, transient assembly was only observed within a narrow window of fuel concentrations, generating small metastable clusters comprising just a few NPs. Higher fuel concentrations irreversibly produced micrometer-sized aggregates (Figure 19B).^262^ This kinetically controlled pathway dependence presumably arises from the stronger van der Waals attractive forces between heavy element cores compared to silicon cores of a similar size.^264^

While dissipative transient assemblies mirror the function of some biological structures, most famously the actin network, several NP properties are suppressed in aggregated states. Transient dispersed states are therefore equally interesting.

Phase cycling between a solid ground state and transient water-dispersed state was achieved for AuNPs coated with aldehyde-terminated ligands, powered by the dissipative consumption of formaldehyde by bisulfite anions (Figure 19C,D).^263^ Using NMR spectroscopy it was possible to unambiguously observe formation of charged α-hydroxysulfonate ligand functional groups, which are responsible for interparticle repulsive forces that sustain the transient dispersed state.^265^ By gradually introducing the formaldehyde ‘fuel’ through acid-catalyzed decomposition of a precursor reservoir—hexamethylene tetramine (HMTA)—the lifetime of the α-hydroxysulfonic acid ligand structure depends on pH,^263^ raising the possibility of using protons as a universal chemical messenger to couple NP assembly state to other chemical processes.

In the long term, multiple reaction cycles operating simultaneously to control both attractive and repulsive forces might generate dissipative structures of far greater structural and functional sophistication, including transient conversion between structurally distinct assembled states. An important step in this direction will be to construct transient superstructures using directional linkages (Figure 15A.1), which would introduce nanocomponent selectivity into the assembly processes.

Overall, by customizing the surface-bound ligands to incorporate directional, high-fidelity molecular recognition motifs or chemospecific covalent reaction sites, it is possible to define atomic-level connectivity between NP building blocks of different types and achieve compositional control. With a multitude of selectively addressable molecular recognition pairs,^266^ orthogonal and complementary dynamic covalent,^267–269^ and coordination bond^270,271^ linkages, spanning a range of equilibration time scales^272^ and compatible with a wide variety of environmental conditions, it can be anticipated that such principles can be further extended as rational strategies for bottom-up construction of multicomponent NP superstructures.

Although chemospecific design strategies have tended to start from extending pairwise interactions that also operate between well-dispersed small-molecule analogues, the inescapable influence of the SAM@NP environment is evident in both experimental and computational studies. Future chemospecific assembly strategies will undoubtedly look to more strongly exploit cooperative features that are unique to the monolayer environment. It is equally possible to generate dynamic assemblies by incorporating addressable functionality in the structures of molecular mediators that interact with SAM@NP ligand shells^20,273,274^ and so systems combining molecular and nanoscale responsive components should be feasible. Another intriguing concept is the potential for catalytically active particles to form dynamic and motile assemblies under the action diffusiophoretic forces, which has been widely studied theoretically and experimentally in microscale colloidal systems.^275–280^ Finally, it must be considered that, for many technological applications, scalability of the procedures and materials remains a considerable challenge, perhaps requiring alternative processing methods to be considered.^194,196,197,281^

## 6 Conclusions and Outlook

Living biological systems excel at producing the right material, at the right time in the right place, with exquisite control over structure across multiple length-scales. Replicating such capabilities in artificial technology is expected to unlock an entirely new class of possibilities in devices, materials and networked chemical systems.^282–286^ Biology exemplifies that nanometer-sized building blocks are essential for processing information encoded in molecular structure and for enabling modular combination of multiple components to produce structural materials, functional assemblages and compartmentalized chemical systems.^287^ Programmable colloidal NPs can be instrumental building blocks and functional components for artificial technologies that exhibit similar capabilities.^288^

Indeed, functionalizing the metal core of colloidal NPs with a self-assembled monolayer provides a highly controllable and versatile handle for creating functional nanosystems. This approach capitalizes on their inherent ability to concentrate small molecules, form distinct populations, catalyze chemical transformations within confined spaces, and respond to external triggers if properly informed. Ultimately, these capabilities converge to support the development of multifunctional architectures with dynamic and adaptive properties.

We highlight that the elegant and versatile concept of multifunctional NPs as powerful controllers of (bio)­systems can offer distinct advantages over classical molecular design approaches. Incorporating catalytic activity, appropriate matrix compatibility and switchability all into a single molecule would be a daunting proposition, whereas making NPs combining all these functions can be achieved by adsorbing on the particles a mixture of relatively simple organic ligands—each possessing one function—in well-chosen proportions. In this spirit, we envisage NPs presenting multiple surface chemistries: some interactions could be responsible for binding to desired targets, some could be switchable to control the nature of interactions in time or in response to external triggers, some could be used to transduce chemical signals, and others could ensure appropriate solubility or biocompatibility. It would be desirable to have the ability to localize these components to discrete regions so that interactions becomes directional^289^ or the effects of interligand cooperativity can be maximized. The chemistry to make multicomponent ligand shells is already relatively well established. As discussed in this Review, there are emerging examples of how to localize different chemical functionalities to different locations. Natural systems may serve as inspiration and learning how to assemble different units at the surface of a single NP may lead to artificial mimics of functional cellular surfaces or intracellular organelles. One could imagine assembling a series of sequential catalysts at a SAM@NP, and exploiting their spatial proximity while minimizing alternative short-circuiting paths. Such functional systems would be reminiscent—as an example—of the respiratory chains in living systems, contributing to the advancement of life-inspired nanomaterials. So, we envisage that spatially organized multifunctional SAM@NP ligand shells will add new dimensions to the capabilities of the systems described throughout this Review.

With a multitude of adsorption sites on the NP surface and the multivalent nature of the monolayer, future developments in monolayer-modulated catalysis may encompass other types of chemical selectivity, including enantiodivergence, control of sequential transformations by turning individual reaction steps on and off, as well as up- and down-regulation of chemical processes occurring simultaneously.

With a wide variety of photoswitchable molecules responsive to different wavelengths of light available, ionizable groups with various p*K*_*a*_ values, or compounds having diverse redox potentials, one could start constructing systems of multiple particles harboring different catalysts. The selective assembly/disassembly of these components—controlled by light of different wavelengths, pH changes or applied potential—could then dictate the sequences in which reactions are catalyzed. Depending on which catalysts are activated at what times, one could synthesize different products from the same initial pool of substrates. Instead of responding to a single trigger (change in temperature, light wavelength, pH, ionic strength) in a rather linear fashion, materials incorporating nanosystems should be able to process multiple inputs to produce programmable signals and other outputs. Chemically responsive and nonequilibrium assembly mechanisms also present the potential of introducing feedback control over SAM@NP catalyst activity.

Furthermore, preformed recognition-enabled and reaction-enabled SAM@NPs provide solution-processable nanoscale building blocks for constructing superstructures on demand, in response to specific chemical or physical stimuli. Using dynamic molecular length-scale interactions confers adaptive behaviors—and, hence, properties—on the resulting assemblies. As hybrid structures themselves, the modular combination of SAM@NPs offers vast structural and compositional variability, giving access to materials properties that derive from structuring on multiple length scales. Predictable construction strategies, underpinned by mechanistic understanding, will consequently define an era of synthetic materials chemistry that is equally adept at working with reaction- and recognition-enabled NP building blocks as it is with molecular synthons.

Several of the studies discussed here have already established that, given sufficient information regarding the underlying molecular-level events, it is possible to achieve a predictive understanding of SAM@NP functional behavior. Yet, quantitatively characterizing molecular length-scale events in nanoconfined environments remains experimentally challenging. A systems-level analysis is required to link the molecular and nanometric features, which can be unique to each NP building block or even each batch of ostensibly the same nanoscale components. One major challenge is the precise characterization of mixed-ligand coatings, which, unlike proteins of similar complexity, cannot be easily crystallized for structural determination via diffraction methods. Additionally, predicting the final monolayer structure and controlling the resulting properties from self-organization, especially when these properties are local and heterogeneous, remain significant obstacles.^290^

Despite the complexity of functional SAM@NPs, advanced computational approaches are increasingly capable of translating theory through to simulations that provide predictive insights on SAM@NP structure, behavior, and assembly pathways, helping to interpret experimental evidence and presenting new hypotheses for experimental testing.^291^ It is therefore realistic to envisage a future where computational tools inform the design and selection of ligands, nanoscale building blocks, and experimental conditions, enabling controlled SAM@NP functioning as well as assembly into superstructures with targeted properties.^292^

## △

Center for Advanced Technologies, Adam Mickiewicz University, Uniwersytetu Poznaskiego 10, Poznań, 61-614, Poland

## Vocabulary

Adaptability: the ability of a chemical system to change its properties as a result of changing environmental conditions, in a path-dependent manner
nanoconfinement: restriction of molecules or physicochemical phenomena within nanoscale spaces
nanozyme: synthetic nanosystem with enzyme-like abilities
programmed self-assembly: organization of building blocks into energy-minimizing structures encoded in the selectivity and directionality of multiple interactions
catalysis regulation: control or modulation of a catalytic activity, selectivity, or stability through external factors or local substrate concentration
suprazyme: supramolecular ensemble of a macrocycle acting as a molecular enzyme mimic and an organic-coated metal NP functioning as a nanozyme
switchability: ability to undergo reversible transitions between distinct states in response to external stimuli.

## Abbreviations

β-CD: β-Cyclodextrin
β-CD-AuNP: β-Cyclodextrin functionalized gold nanoparticle
β-CD-SiO_2_: β-Cyclodextrin functionalized silica nanoparticle
3-MT: 3-Methoxythreonine
AB: Azobenzene
AgNP: Silver nanoparticle
Alloc: Allyloxycarbonyl
AMP: Adenosine monophosphate
ATP: Adenosine triphosphate
ATPF: 2-Aminopurine riboside-5′-*O*-triphosphate
Au@AB: AB-functionalized Au nanomaterials
AuNP: Gold nanoparticle
CBPQT: Cyclobis­(paraquat-*p*-phenylene)
cod: cycloocta-1,5-diene
Cp: pentamethylcyclopentadienyl
CPMGz: Carr–Purcell–Meiboom–Gill with a *z*-gradient
cyclen: 1,4,7,10-Tetraazacyclododecane
DASA: Donor–acceptor Stenhouse adduct
DCNPs: Dynamic covalent nanoparticles
dma-AB: *N*,*N*-Dimethylamine-substituted azobenzene
DNP: Dioxynaphthalene
DOSY: Diffusion ordered spectroscopy
EDC: 1-Ethyl-3-(3-(dimethylamino)­propyl)­carbodiimide
EDU: 1-Ethyl-3-(3-(dimethylamino)­propyl)­urea
EGFP: Enhanced green fluorescent protein
ESR: Electron spin resonance
GMP: Guanosine monophosphate
HMTA: Hexamethylene tetramine
HPNP: 2-Hydroxypropyl p-nitrophenyl phosphate
IDA: Indicator displacement assay
ITO: Tin-doped indium oxide
Le^X^: Trisaccharide Lewis X
M: Monomer
MD: Molecular dynamics
MUA: 11-Mercaptoundecanoic acid
NHS: *N*-Hydroxysuccinimido
NMR: Nuclear magnetic resonance
NP: Nanoparticle
NOE: Nuclear Overhauser effect
PDMS: Polydimethylsiloxane
Pi: Inorganic phosphate
RBCs: Red blood cells
SAM: Self-assembled monolayer
SAM@NPs: Self-assembled monolayer-protected nanoparticles
SSC: Surface saturation concentration
SOAP: Smooth overlap of atomic positions
SPR: Surface plasmon resonance
STD: Saturation transfer difference
T: Thymine
TACN: Triazacyclononane
TDP: Thymine diphosphate
TMCs: Transition metal complexes
TMP: Thymidine monophosphate
TPEN: *N*,*N*,*N*′,*N*′-tetrakis­(2-Pyridylmethyl)­ethylenediamine
TREN-Im: Imidazole-functionalized tris­(2-Aminoethyl)­amine
TTF: Tetrathiafulvalene
UV: Ultraviolet
